# *Tbx15* controls skeletal muscle fibre-type determination and muscle metabolism

**DOI:** 10.1038/ncomms9054

**Published:** 2015-08-24

**Authors:** Kevin Y. Lee, Manvendra K. Singh, Siegfried Ussar, Petra Wetzel, Michael F. Hirshman, Laurie J. Goodyear, Andreas Kispert, C. Ronald Kahn

**Affiliations:** 1 Section on Integrative Physiology and Metabolism, Joslin Diabetes Center, Harvard Medical School, 1 Joslin Plaza, Boston, Massachusetts 02215, USA; 2 Institut für Molekularbiologie, Medizinische Hochschule Hannover, Carl-Neuberg-Str. 1, D-30625 Hannover, Germany; 3 Signature Research Program in Cardiovascular and Metabolic Disorders, Duke-NUS Graduate Medical School Singapore, National Heart Centre Singapore, 8 College Road, Singapore 169857, Singapore; 4 Institute for Diabetes and Obesity, Helmholtz Center, Parkring, 1385748 Munich/Garching, Germany; 5 Zentrum Physiologie, Medizinische Hochschule Hannover, Carl-Neuberg-Str. 1, D-30625 Hannover, Germany

## Abstract

Skeletal muscle is composed of both slow-twitch oxidative myofibers and fast-twitch glycolytic myofibers that differentially impact muscle metabolism, function and eventually whole-body physiology. Here we show that the mesodermal transcription factor T-box 15 (*Tbx15*) is highly and specifically expressed in glycolytic myofibers. Ablation of *Tbx15 in vivo* leads to a decrease in muscle size due to a decrease in the number of glycolytic fibres, associated with a small increase in the number of oxidative fibres. This shift in fibre composition results in muscles with slower myofiber contraction and relaxation, and also decreases whole-body oxygen consumption, reduces spontaneous activity, increases adiposity and glucose intolerance. Mechanistically, ablation of *Tbx15* leads to activation of AMPK signalling and a decrease in *Igf2* expression. Thus, *Tbx15* is one of a limited number of transcription factors to be identified with a critical role in regulating glycolytic fibre identity and muscle metabolism.

## Abstract (web summary)

The transcriptional regulator Tbx15 has a role in organ development. Here Lee *et al.* show that Tbx15 influences fibre-type determination in murine skeletal muscles, explaining local and systemic metabolic derangements in heterozygous Tbx15 knockout mice.

## Graphical abstract

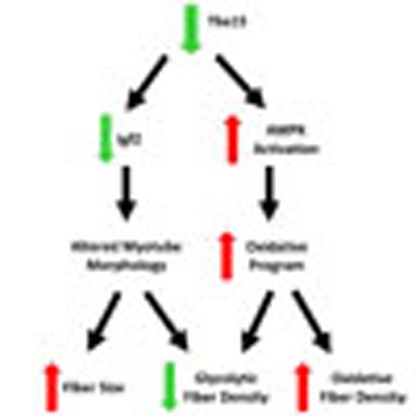

The association of type 2 diabetes mellitus with obesity and inactivity illustrates the important link between energy homeostasis and the development of metabolic disease. Skeletal muscle, because of its high oxidative capacity and large contribution to total body mass, is an important tissue in maintaining normal whole-body metabolism and energy homeostasis.

In mammals, skeletal muscles are composed of a mosaic of different fibre types. Slow-twitch (type I) myofibers are rich in the mitochondria, have high oxidative capacity and high capillary density, whereas fast-twitch (type II) fibres have lower mitochondrial and capillary density and generate ATP primarily through glycolysis. A number of studies have demonstrated that individuals with type 2 diabetes mellitus or obesity have more glycolytic fibres and less oxidative fibres than healthy individuals^1^,^2^,^3^,^4^. Indeed, fibre-type distribution has been shown to directly correlate with glucose uptake and insulin resistance in humans^5^.

The molecular regulation of fibre-type determination is not completely understood. However, recent studies have begun to identify some of the pathways that interact to affect fibre-type distribution. For example, pathways that drive the formation of oxidative myofibers include those mediated by AMP-activated protein kinase (AMPK), peroxisome proliferator-activated receptor gamma co-activator 1-alpha (PGC-1α), calcineurin and protein kinase C^6^,^7^,^8^,^9^,^10^. On the other hand, glycolytic muscle determination is, at least in part, controlled by the transcriptional co-activator Baf60c and involves activation of Akt through the mTor-interacting protein Deptor^11^.

*Tbx15* is a member of the T-box gene family that contains 17 different members in mammals. The first T-box gene characterized was Brachyury (T), a gene known to play a major role in mesodermal development in all vertebrates^12^. The T-box transcription factors share a characteristic sequence similarity within the DNA-binding domain (T-domain) that is involved in control of a variety of developmental processes^13^, including mesoderm specification, somite segmentation and left/right body axis determination^14^. Complete inactivation of the *Tbx15* gene in mice and mutations of *TBX15* in humans result in severe skeletal malformation^15^,^16^,^17^. Depending on cellular context, Tbx15 can activate gene transcription, or through its interactions with co-repressors of the Groucho family, repress gene transcription^18^.

Work from our lab has demonstrated that *Tbx15* is differentially expressed between different adipose tissue depots, and its expression in human visceral adipose depots is strongly downregulated in overweight and obese individuals^19^. In addition, genome-wide association studies have shown that single-nucleotide polymorphisms within the *TBX15* gene correlate independently with both body mass index and waist–hip ratio, a measure of central obesity^20^. *In vitro*, we have shown that Tbx15 negatively regulates mitochondrial mass and activity in pre-adipocytes, suggesting a role in the control of oxidative versus glycolytic metabolism^21^. Consistent with this, *Tbx15* was recently also identified in a panel of genes that were more highly expressed in glycolytic than oxidative muscle^11^.

The ability of Tbx15 to regulate mitochondrial mass and activity, its high expression in glycolytic muscle, and the correlation of both polymorphisms and expression of *Tbx15* to obesity lead us to hypothesize that Tbx15 might regulate metabolic function and fibre type in skeletal muscle. In the current study, we show that Tbx15 is specifically expressed in glycolytic muscle fibres. Ablation of *Tbx15* leads to a decrease in glycolytic fibres and increase in oxidative fibres. This leads to the generation of skeletal muscle with increased oxidative fibre density and slow contractile properties. Loss of Tbx15 also causes a dose-dependent increase in fibre size. Heterozygous deletion in mice (*Tbx15*^*+/−*^) results in a milder shift in fibre type, increased adiposity and glucose intolerance. *In vitro* studies indicate that Tbx15 acts through downregulation of the AMPK signalling pathway and *Igf2*, and exogenous administration of AMPK inhibitors or recombinant insulin growth factor 2 (Igf2) partially rescues the metabolic phenotypes and abnormal morphology of *Tbx15* knockdown myotubes. Taken together, these data indicate that Tbx15 has a critical role in regulating muscle fibre type, muscle metabolism and whole-body physiology.

## Results

### Tbx15 is highly and specifically expressed in glycolytic myofibers

**Figure 1:**
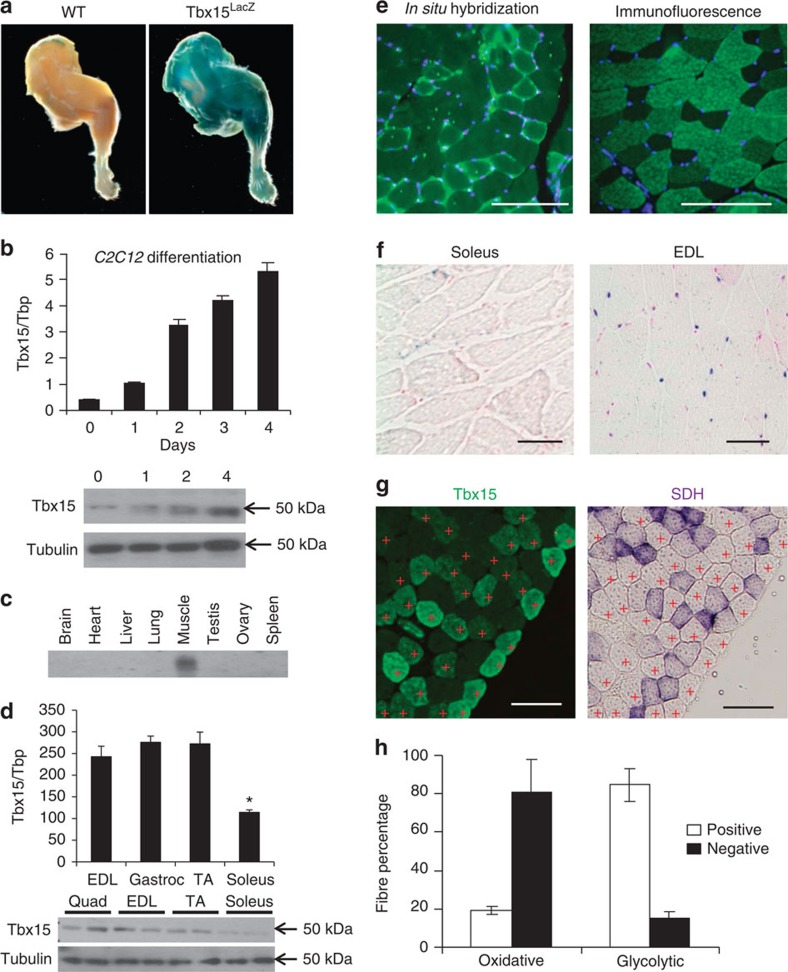
Tbx15 is highly and specifically expressed in glycolytic skeletal muscle. (**a**) X-gal-stained hind limbs from 3-month-old *Tbx15*^*LacZ/+*^ males. (**b**) qPCR analysis of *Tbx15* expression from C2C12 cells during myogenic differentiation. Data are shown as mean±s.e.m. of triplicate samples and repeated three times (upper panel). Western blot of Tbx15 from protein extracts from the same cells using tubulin as a loading control (lower panel). (**c**) Expression level of *Tbx15* mRNA was compared by northern blot of RNA isolated from tissues from of 8-week-old male and female (ovary) C57BL/6 mice. This experiment has been performed once. (**d**) qPCR analysis of *Tbx15* expression from muscle groups of 8-week-old male C57BL/6 mice. Data are shown as mean±s.e.m. of six samples (upper panel). Western blot of Tbx15 from protein extracts made from the same muscles using tubulin as a loading control (lower panel). (**e**) Fluorescent *in situ* hybridization for *Tbx15* in quadriceps muscle and immunofluorescence for Tbx15 in tibialis anterior muscle of 8-week-old random fed male mice. The photographs were taken at × 10 magnification. The photographs were taken at × 10 magnification. Scale bar, 100 μM. (**f**) X-gal-stained representative sections of soleus and EDL from 8-week-old *Tbx15*^*LacZ*^ males (*n*=3). Slides were lightly counterstained with eosin. Pictures were taken at × 20 magnification. Scale bar, 50 μM. (**g**) Immunofluorescence for Tbx15 and succinate dehydrogenase staining was performed on serial sections of tibialis anterior muscle of 8-week-old random fed male mice. Glycolytic fibres are marked with red crosses. The photographs were taken at × 10 magnification. Scale bar, 100 μM (**h**) Immunofluorescence for Tbx15 and succinate dehydrogenase staining was performed on serial sections. Five digital images (× 20) from non-overlapping fields were taken from each slide (total 20 fields per group), and oxidative and glycolytic muscle fibres were scored for Tbx15 expression. Values are mean±s.e.m. of four animals.

*Tbx15* is a mesodermal gene involved in normal skeletal and muscle development^17^. In *Tbx15*^*LacZ/+*^ mice, in which the *LacZ* gene replaced exon 3 of the *Tbx15* gene creating a fusion protein allowing tracking of *Tbx15* expression^17^, there was robust X-gal staining in the skeletal muscle with no staining in the control (Fig. 1a). In C2C12 myoblasts, expression of *Tbx15* messenger RNA (mRNA) increased ∼12-fold during differentiation, and this was confirmed by western blot analysis (Fig. 1b). Northern blot and quantitative PCR (qPCR) analysis of mouse tissues revealed that *Tbx15* expression was at least eight times higher in muscle than adipose tissue, skin and pancreas, with much lower levels in other tissues (Fig. 1c; Supplementary Fig. 1a).

Assessment of individual muscles showed that *Tbx15* mRNA was over two-fold higher in glycolytic muscles, such as the extensor digitorum longus (EDL), gastrocnemius and tibialis anterior, than in the oxidative muscle, such as soleus; and this was confirmed by western blot analysis (Fig. 1d). Fluorescent *in situ* hybridization on quadriceps muscle of wild-type mice demonstrated that *Tbx15* message was not uniformly expressed, but in a mosaic pattern of fibre types. Similarly, immunofluorescence for Tbx15 in tibialis anterior muscle, which contains a mixture of oxidative and glycolytic fibres, confirmed that Tbx15 was expressed in a fibre-type-specific manner (Fig. 1e).

**Figure 2:**
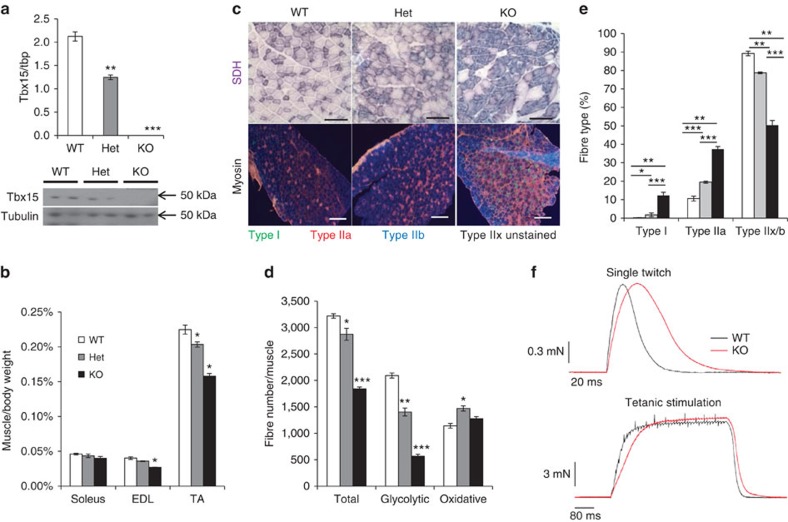
Ablation of *Tbx15* increases oxidative fibre density, reduces muscle mass and increases fibre diameter. (**a**) qPCR analysis for *Tbx15* mRNA of RNA isolated from the tibialis anterior of male wild-type (WT), *Tbx15*^*+/−*^ and *Tbx15*^*−/−*^ mice at 6–8 weeks of age. Data are shown as mean±s.e.m. of three to eight animals per group. Asterisks indicate significant differences in all panels. (*P<0.05; **P<0.01; ***P<0.001 by analysis of variance). (**b**) Mass of soleus, extensor digitorum longus (EDL) and tibialis anterior (TA) of male WT, *Tbx15*^*+/−*^ and *Tbx15*^*−/−*^ mice at 6–8 weeks of age normalized to body weight. Data are shown as mean±s.e.m. of three to eight animals per group. (**c**) Succinate dehydrogenase (SDH) staining (top panels) from tibealis anterior and immunofluorescence for myosin I, IIa and IIb (bottom panels) from WT, *Tbx15*^*+/−*^ and *Tbx15*^*−/−*^ EDL muscle. SDH pictures are taken at × 20. Scale bar, 100 μM. Myosin immunofluorescence pictures are taken at × 10. Scale bar, 200 μM. (**d**) Quantitation of glycolytic and oxidative muscle fibres from the rectus femoris quadriceps muscle of male WT, *Tbx15*^*+/−*^ and *Tbx15*^*−/−*^ mice at 6–8 weeks of age. Data are shown as mean±s.e.m. of three to eight animals per group. (**e**) Quantitation of fibre types from the EDL muscle of male WT, *Tbx15*^*+/−*^ and *Tbx15*^*−/−*^ mice at 6–8 weeks of age. Data are shown as mean±s.e.m. of three to four muscles per group. (**f**) Representative recordings of single-twitch contraction and tetanic stimulation of EDL fibre bundles from WT and *Tbx15*^*−/−*^ mice at 3–4 months of age (*n*=8–10).

To determine which specific fibres exhibited high Tbx15 expression, we stained individual muscles from *Tbx15*^*LacZ*^ mice. As shown in Fig. 1f, Tbx15-related X-gal staining was observed only in the glycolytic EDL muscle and not in the oxidative soleus muscle. When sections from tibialis anterior muscle were double stained using antibodies to Tbx15 and myosin IIa, it was clear that Tbx15 was virtually absent from type IIa, fast-twitch, oxidative fibres (Supplementary Fig. 1c). Furthermore, when serial sections were stained for succinate dehydrogenase (SDH), a marker of mitochondrial activity, <20% of the oxidative fibres, but over 80% of the glycolytic muscle fibres, scored positive for Tbx15 expression (Fig. 1g,h). Taken together, these data indicate that Tbx15 is expressed almost exclusively fast-twitch, glycolytic muscle. Interestingly, immunofluorescence also showed that Tbx15 was expressed in both the cytoplasm and nuclei of muscle fibres. These findings were confirmed by cell fractionation studies of skeletal muscle, which showed Tbx15 was present in the cytoplasm, microsomal fraction, and the nucleus (Supplementary Fig. 1b). Antibody specificity was verified by the absence of positive signal in both western blot analysis and immunofluorescence of Tbx15 in *Tbx15*^*−/−*^ muscle (Fig. 2a; Supplementary Fig. 1d).

Endurance exercise in both humans and mice has been shown to lead to an increase in oxidative and decrease in glycolytic fibre density^22^. qPCR analysis revealed that 3 weeks of voluntary wheel cage running reduced mRNA Tbx15 levels by 15% in 6-week-old wild-type (WT) males. This is consistent with a reduction in glycolytic fibres, as myosin IIb mRNA levels were reduced 19%. Other markers of oxidative metabolism, including Pgc-1α, SDH, as well as markers of oxidative fibres, myosin I and myosin IIa, were all increased in exercised muscle (Supplementary Fig. 1e).

### Ablation of Tbx15 leads to a decrease in glycolytic myofibers

To define the role of *Tbx15* in skeletal muscle, we studied homozygous (*Tbx15*^*−/−*^) and heterozygous *Tbx1*5 knockout (*Tbx15*^*+/−*^) mice. As previously reported, homozygous *Tbx15* knockout animals had shortened limbs and other skeletal malformations^17^, whereas overt developmental abnormalities were noted in the heterozygous knockout animals. qPCR and western blot analysis of quadriceps muscle at 6 weeks of age shows that *Tbx15* expression was reduced by about ∼40% in the heterozygous knockout animals and completely abrogated in the homozygous knockout (Fig. 2a). Despite the lack of gross abnormalities, heterozygous ablation of *Tbx15* led to a significant ∼10% reduction in muscle mass (even when normalized to body weight) of both the EDL and tibialis anterior muscles, which are comprised mainly of glycolytic fibres. By contrast, there was no change in the weight of the more oxidative soleus muscle. The reduction in muscle mass was even more marked in *Tbx15*^*−/−*^ mice with a 20–25% reduction in weight of the EDL and tibialis anterior muscles, again with no changes in the soleus muscle weight (Fig. 2b).

On histological examination of tibialis anterior from 6-week-old animals, there was a moderate increase in the relative density of oxidative, SDH positive fibres in the heterozygous knockout mice and a marked increase in the homozygous knockout compared with controls, in both cases with a corresponding decrease in glycolytic, SDH negative fibres (Fig. 2c). Quantitation of fibres from SDH-stained quadriceps muscle revealed a reduction in the total number of fibres of 11% in heterozygous and 43% in homozygous knockout mice. This reduction was due to a specific and gene dose-dependent reduction in the number of glycolytic muscle fibres of 33% in heterozygous and 72% in homozygous knockout mice (Fig. 2d). On the other hand, the number of oxidative fibres was significantly increased by 28% in heterozygous and was also increased by 12% in the muscle of homozygous knockout mice, although the latter did not quite reach statistical significance (analysis of variance; *P*=0.1).

Muscle fibre-type assessment by immunofluorescent staining for myosin heavy chain isoforms revealed that ablation of Tbx15 led to the appearance of considerable numbers of type I fibres in *Tbx15*^*+/−*^ and *Tbx15*^*−/−*^ EDL compared with WT mice, which have virtually no type I fibres in EDL muscle (Fig. 2c). Furthermore, these EDL muscles had a 2–3.5-fold increase in type IIa fibre density compared with controls. This increase in oxidative fibre density is due to a transformation of fibres into oxidative type I and type IIa fibres, as well as a reduction in glycolytic IIb fibres by 12 and 44% in the *Tbx15*^*+/−*^ and *Tbx15*^*−/−*^ EDL, respectively (Fig. 2e). Interestingly, despite the reduction in muscle weight being caused by a reduction in the number of glycolytic fibres in *Tbx15*^*+/−*^ and *Tbx15*^*−/−*^ mice, the cross-sectional area (CSA) of the remaining glycolytic fibres was significantly increased by 13 and 46% in tibialis anterior muscles of *Tbx15*^*+/−*^ and *Tbx15*^*−/−*^ animals, respectively. CSA of oxidative fibres was also increased by 18 and 57% in tibialis anterior muscles of *Tbx15*^*+/−*^ and *Tbx15*^*−/−*^ animals, respectively (Supplementary Fig. 2a). These changes in fibre size were associated with a change and broadening of distribution, as reflected in the s.d. of fibre size within each animal. This is true for both the glycolytic and oxidative fibres of *Tbx15*^*+/−*^ and *Tbx15*^*−/−*^ muscles, respectively (Supplementary Fig. 2b–c).

### Ablation of Tbx15 leads to a decreased rate of muscle contraction

Since muscle fibre-type controls contraction and relaxation rates of skeletal muscle, with oxidative fibres characterized as slow-twitch and glycolytic fibres as fast twitch, we analysed the impact of loss of *Tbx15* on muscle contractile properties. Both twitch and tetanic contraction were tested *ex vivo* using small fibre bundles of the EDL, normally a fast-twitch muscle. Recordings of single twitches were analysed for peak force, time to peak (TTP), and 50 and 75% relaxation times (t50 and t75%; Fig. 2f; Supplementary Fig. 2d). During single-twitch contraction, peak force was not altered, however. the rate of contraction, as measured by time to peak, was prolonged by ∼30% in *Tbx15*^*−/−*^ muscles characteristic of slow, oxidative fibres. Likewise, muscle relaxation, as indicated by increased t50 and t75% values, was retarded in *Tbx15*^*−/−*^ mice by ∼35%. After tetanic stimulation, muscle fibres of *Tbx15*^*−/−*^ mice also demonstrated a ∼30% slower relaxation rate, as indicated by increase t50 and t75% values, compared with WT, again with no change in peak force (Supplementary Fig. 2d).

### Ablation of Tbx15 does not affect skeletal muscle performance

To test whether the ablation of Tbx15 led to changes in muscle performance, we performed several tests of muscle performance both *ex vivo* and *in vivo*. In agreement with the results found in *Tbx15*^*−/−*^ mice, no differences in maximal twitch force or tetanic force were observed in EDL muscle from *Tbx15*^*+/−*^ mice compared with WT controls (Supplementary Fig. 2e–f). There was also no difference in fatigue resistance, as assessed by reduction in maximal tetanic force after repeatedly stimulated (Supplementary Fig. 2g).

In a treadmill exercise paradigm on 8-week-old male mice, there was no significant difference in the running time (13.6±1.5 min for WT versus 13±1.1 min for Tbx15^+*/−*^, *n*=6–8) or the total distance run (126.5±21.5 m versus 122.5±10.7 m; Supplementary Fig. 2h). Likewise, no differences were observed in grip strength (67.3±3.0 versus 62.8±3.1 g, *n*=12–14; Supplementary Fig. 2i). Finally, although two separate cohorts of Tbx15^+*/−*^ males tended to show less voluntary wheel running than control mice (8.06±0.70 km per day for WT versus 7.16±0.61 km per day for Tbx15^+*/−*^, *n*=6), this was not statistically significant (Supplementary Fig. 2j). Thus, even though Tbx15 ablation caused both cellular and molecular changes in skeletal muscle, standard tests of muscle performance were not significantly affected.

### Tbx15 ablation causes glucose intolerance and obesity

**Figure 3:**
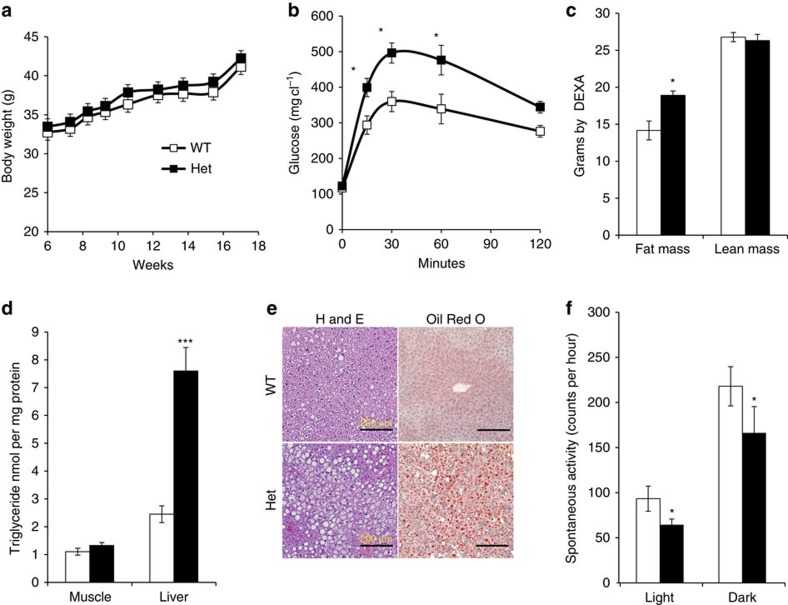
*Tbx15*^*+/−*^ males are resistant to high-fat diet-induced obesity. (**a**) Body weights of *Tbx15*^*+/−*^ and control males during 10 weeks on high-fat diet or chow diet (started at 6 weeks of age). Data are shown as mean±s.e.m. of 10–12 animals per group. (**b**) Glucose tolerance testing of 5-month-old *Tbx15*^*+/−*^ and control males. Data are shown as mean±s.e.m. of 10–12 animals per group (*P<0.05 for all panels by Student’s *t*-test or Mann–Whitney *U* statistical tests). (**c**) Lean and fat mass from 6-month-old *Tbx15*^*+/−*^ and control males. Data are shown as mean±s.e.m. of six to seven animals per group. (**d**) Triglyceride quantitation from liver and muscle extracts of 6-month-old *Tbx15*^*+/−*^ and control males. Data are shown as mean±s.e.m. of six animals per group. (**e**) Haematoxylin and eosin staining (left panels) and Oil Red O staining (right panels) of 6–month-old *Tbx15*^*+/−*^ and control livers. Nuclei are counterstained with haematoxylin. Representative digital images (× 20) are shown. Scale bar, 200 μM. (**f**) Spontaneous activity the light and dark cycles of pads from 5-month-old *Tbx15*^*+/−*^ and control males. Data are shown as mean±s.e.m. of six animals per group.

Factors that affect muscle fibre-type distribution and size can lead to alterations in whole-body physiology and metabolism^22^,^23^. Since homozygous *Tbx15* knockout animals exhibited obvious developmental abnormalities that might also affect metabolic physiology, we focused our assessment on developmentally normal, heterozygous *Tbx15* knockout animals. At 5 months of age, *Tbx15*^*+/−*^ mice fed a standard chow (21% fat by calories) exhibited no differences from WT in body weight, fed or fasting blood glucose, and circulating insulin levels (Fig. 3a; Supplementary Fig. 3a–b). However, when challenged with an intraperitoneal glucose tolerance test, 5-month-old *Tbx15*^*+/−*^ mice showed impaired glucose tolerance with a 43% increase in peak glucose levels (Fig. 3b). This occurred with no change in insulin tolerance, or insulin levels during the intraperitoneal glucose tolerance test (Supplementary Fig. 3c–d). At this age, Dual-energy X-ray absorptiometry (DEXA) analysis revealed that *Tbx15*^*+/−*^ mice had no change in lean mass, but a 21% increase in fat tissue mass compared with controls (Fig. 3c). Haematoxylin/eosin staining, Oil Red O staining and biochemical analysis of the livers from 6-month-old *Tbx15*^*+/−*^ animals also revealed a threefold increase in hepatic lipid accumulation, with no increase in muscle triglyceride content (Fig. 3d,e).

Indirect calorimetry of 5-month-old mice revealed that *Tbx15*^*+/−*^ males had ∼12% decrease in average oxygen consumption during both the light phase (3,386+174 versus 3,038+75 ml kg^−1^ lean mass per hour in control versus *Tbx15*^*+/−*^ mice) and dark phase (3,049+120 versus 2,688±53 ml kg^−1^ lean mass per hour) of the diurnal cycle (Supplementary Fig. 3d) with no change in respiratory quotient (Supplementary Fig. 3e). Although the differences in oxygen consumption did not quite reach statistical significance (Student’s *t*-test; *P*=0.1), similar trends were observed in three separate cohorts of mice. This tendency of decreased oxygen consumption was associated with a significant 25–30% decrease in spontaneous activity of *Tbx15*^*+/−*^ mice during both the light phase (93±8 versus 64±5 counts per hour in control versus *Tbx15*^*+/−*^ mice per hour) and dark phase (217±21 versus 166±29 counts per hour) of the diurnal cycle (Fig. 3f). Since Tbx15 is almost exclusively expressed in skeletal muscle, with no detectable expression in the brain during any period in development, the reduction in activity after Tbx15 ablation is presumably due to its effects on the skeletal muscle. Thus, although heterozygous deletion of *Tbx15* does not impair exercise-induced activity, it does result in decreased spontaneous activity that, at least in part, contributes to an increase in adiposity and hepatosteatosis.

### Tbx15 regulates oxidative capacity and AMPK signalling

**Figure 4:**
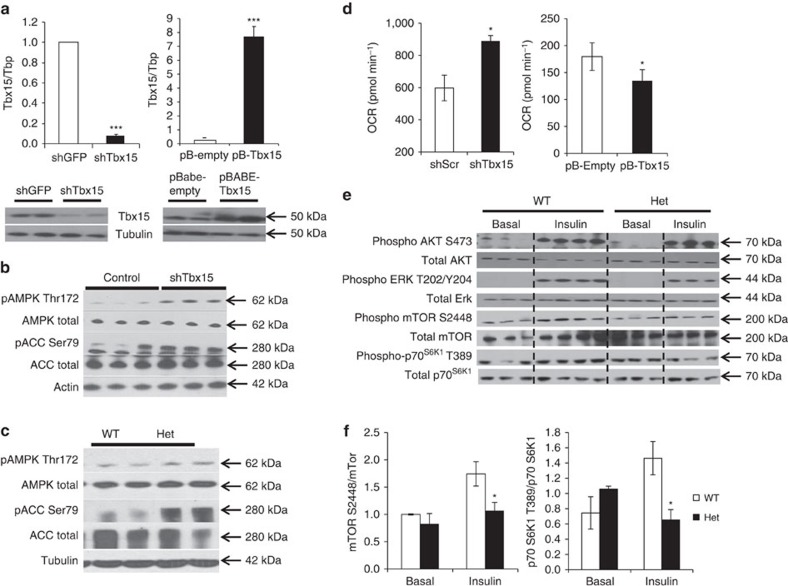
Tbx15 regulates oxidative capacity and AMPK signalling in skeletal muscle. (**a**) Expression of *Tbx15* mRNA and protein were compared by qPCR and western blot analysis between C2C12 myoblasts stably transfected with *shTbx15* or shGFP (control), and C2C12 myoblasts stably transfected with pBABE-*Tbx15* or pBABE-Empty (control). Data shown as mean±s.e.m. of three independently transfected samples and was repeated three times. Western blot of Tbx15 from protein extracts from the same cells. Western blot for tubulin was used as a loading control (*P<0.05; **P<0.01; ***P<0.001 for all panels by Student’s *t*-test). (**b**) Western blot analysis of phosphorylation of AMP kinase (AMPK) at Thr172 and acetyl-CoA carboxylase (ACC) at Ser79 and total protein controls from myoblasts stably transfected with *shTbx15* or controls. Actin is used as a loading control. (**c**) Western blot analysis of phosphorylation of AMPK at Thr172 and Acc1 at Ser79 and total protein controls from skeletal muscle of 6-week-old *Tbx15*^*+/−*^ and control males. Tubulin is used as a loading control. (**d**) Basal respiration of *shTbx15*, pBABE-*Tbx15* and control C2C12 myoblasts was determined by calculating the area under the curve (AUC) during measurements of basal respiration. Values are means±s.e.m. of six to seven replicates. The whole experiment was repeated three times. (**e**) Western blot analysis of phosphorylation of AKT, ERK, mTOR, and p70^SK1^ and total protein controls from tibealas anterior of in 10-week-old *Tbx15*^*+/−*^ and control male mice 5 U of insulin was injected per mouse and tissues were collected 15 min later. (**f**) Quantitation of western blots of phosphorylation of mTOR, and p70^SK1^ and total protein controls in Supplementary Fig. 4a. Values are means±s.e.m. of six to seven replicates.

To further investigate the mechanisms underlying the loss of glycolytic fibres and increase in oxidative fibres observed in the mice with reduced *Tbx15*, we created C2C12 myoblast cell lines with a stable knockdown of *Tbx15* expression (*shTbx15* cells) and compared them with C2C12 cells with stable overexpression of *Tbx15* (pBABE-*Tbx15*). qPCR and western blot analysis demonstrated that *Tbx15* mRNA and Tbx15 protein were reduced >90% in *shTbx15* cells and increased ∼20-fold in pBABE-Tbx15 cells (Fig. 4a).

AMPK signalling has been shown to be both necessary and sufficient to transform glycolytic muscle fibres into oxidative fibres^8^,^10^. Western blot analysis of control and *shTbx15* myoblasts demonstrated a robust increase in phosphorylation of AMPK on Thr172 and its downstream substrate acetyl-CoA carboxylase (ACC) on Ser79 compared with controls (Fig. 4b), with no change in total levels of AMPK or ACC. This was confirmed *in vivo* in extracts of muscle from *Tbx15*^*+/−*^ mice, which showed an increase in AMPK Thr172 and ACC Ser79 phosphorylation (Fig. 4c).

Activation of AMPK signalling usually leads to an enhancement of oxidative metabolism and increased oxygen consumption rates (OCRs)^24^. Indeed, although whole-body oxygen consumption tended to be decreased in *Tbx15*^*+/−*^ mice, knockdown of *Tbx15* in C2C12 cells resulted in a 33±6% increase in basal OCRs, and treatment of the *shTbx15* and control myotubes with the AMPK inhibitor, compound C, reduces OCR in both the groups (Fig. 4d; Supplementary Fig. 4a). Conversely, overexpression of *Tbx15* in C2C12 myoblasts resulted in a decrease in basal OCR by 34±1% compared with controls (Fig. 4d). Thus, reduction of Tbx15 both *in vivo* and *in vitro* leads to an activation of the AMPK signalling pathway. At the cellular level, this leads to an increase in oxidative metabolism in C2C12 myoblasts, but this effect is masked *in vivo*, as a result of the decreased activity of the *Tbx15*^*+/−*^ mice.

AMPK has a well-established role in regulating insulin resistance, and activation of AMPK has been to shown to directly inhibit mTor signalling^25^,^26^. To examine the effects of AMPK activation we observed on ablation of Tbx15, we investigated insulin and mTOR signalling in fasted WT and Tbx15^+*/−*^ muscle 15 min after intravenous administration of insulin. Although insulin stimulation robustly increased phosphorylation of AKT S473 and ERK T202/Y204, no differences in total AKT or ERK proteins or in the phosphorylation of these proteins was observed between WT and Tbx15^+*/−*^ muscle (Fig. 4e). Likewise, qPCR analysis showed no changes in the glycolytic muscle-specific regulators of Akt activation, Baf60c and Deptor^11^ (Supplementary Fig. 4b–c) or other regulators of muscle fibre type, including PGC-1α, PGC-1β, RIP140, calcineurin or Ppar-delta (Supplementary Fig. 5). However, after insulin stimulation, levels of phosphor mTor S2448 as well as its downstream phosphorylation target p70^S6K1^ T389 were significantly reduced in Tbx15^+*/−*^ muscle, with no changes in levels of total mTor or p70^S6K1^ (Fig. 4f). Since AMPK has been shown to repress activation of mTor signalling, these results are consistent with the marked activation of the AMPK signalling axis in Tbx15^+*/−*^ muscle.

### Tbx15 regulates Igf2 levels both *in vivo* and *in vitro*

To determine possible transcriptional targets of Tbx15, we performed microarray analyses on *shTbx15* and pBABE-Tbx15 myoblasts. Differentially regulated genes and pathways were analysed focusing on genes that were oppositely regulated in the *Tbx15* knockdown and overexpressing cells. Genes that were significantly downregulated in *shTbx15* and upregulated in pBABE-*Tbx15* (with fold change >1.5; q<0.10) are found in Supplementary Table 3, and genes that were significantly upregulated in *shTbx15* and downregulated in pBABE-*Tbx15* are shown in Supplementary Table 4. Gene set enrichment analysis and ingenuity pathway analysis of these data revealed that the Igf pathways were among the most significantly altered in the *Tbx15* cellular models. In particular, the expression of the protein hormone *Igf2* showed robust and opposite regulation in these *Tbx15* cellular models.

**Figure 5:**
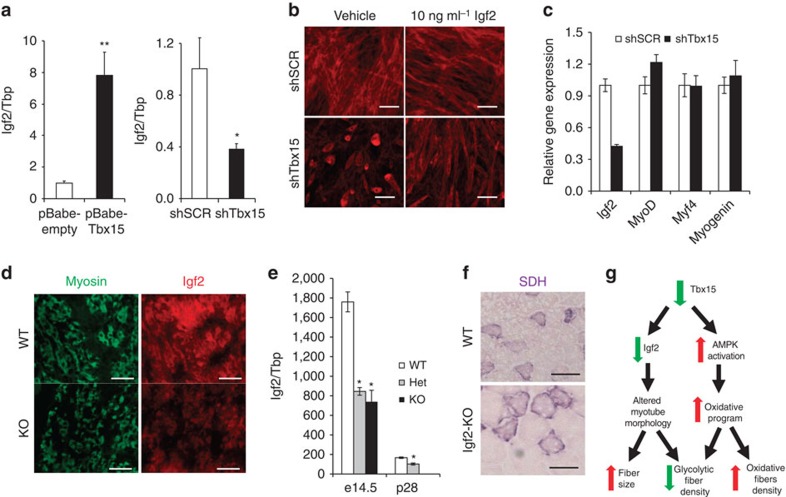
Tbx15 regulates *Igf2* and is critical for myotube formation. (**a**) Expression level of *Igf2* mRNA assessed by qPCR in C2C12 myoblasts stably transfected with pBABE-*Tbx15* and pBABE-Empty (control), and C2C12 myoblasts stably transfected with *shTbx15* and shGFP (control). Data shown as the means±s.e.m.’s of three independently transfected samples (*P<0.05 for all panels by Student’s *t*-test). (**b**) Phalloidin-Alexa-546 staining of *shTbx15* and shGFP (control) myotubes after 4 days of differentiation either treated with vehicle (0.1% BSA) or 10 ng ml^−1^ recombinant Igf2. Pictures were taken at × 10 magnification. Scale bar, 50 μM. (**c**) Expression level of *Igf2*, *MyoD*, *Myf5* and *myogenin* mRNA was assessed by qPCR in C2C12 myoblasts stably transfected with *shTbx15* and shGFP (control) myotubes after 4 days of differentiation. Data are shown as mean±s.e.m. of three independently transfected samples. The experiment was repeated three times. (**d**) Immunofluorescence staining for total myosin isoforms (left) and Igf2 (right) from developing muscles in the limb buds of wild-type (WT) and *Tbx15*^*−/−*^ E14.5 embryos. Pictures are taken at × 20 magnification. Scale bar, 100 μM. (**e**) Expression level of Igf2 mRNA was compared by qPCR in dissected limb muscle from WT, *Tbx15*^*+/−*^ and *Tbx15*^*−/−*^ E14.5 embryos and from tibialis anterior skeletal muscle from at WT and *Tbx15*^*−/−*^ male mice at p28. Values are means±s.e.m. of four to six animals per group. (**f**) Succinate dehydrogenase (SDH) staining of tibialis anterior muscle from 8-week-old WT and Igf2 knockout (*n*=3). Pictures were taken at × 20. Scale bar, 50 μM. (**g**) Model of Tbx15 action in skeletal muscle.

qPCR analysis confirmed that the expression of *Igf2* was increased over sevenfold in *Tbx15* overexpressing cells and was decreased by over 70% in *shTbx15* knockdown cells compared with controls (Fig. 5a). Since Igf2 is an important regulator of myogenesis and myoblast differentiation^27^, we investigated whether *shTbx15* myoblasts that exhibit reduced *Igf2* levels also had a defect in myogenesis. Indeed, compared with control myotubes, after 4 days of differentiation *shTbx15* cells showed a reduced number of myotubes, but those that did form were both shorter and thicker than control differentiated cell (Fig. 5b, left panels). Although markers of myogenesis *MyoD*, *Myf4* and myogenin were normally induced in the knockdown cells, *Igf2* levels remain reduced in the differentiated *shTbx15* myotubes (Fig. 5c). In further support of a combined role for Tbx15 and Igf2 in myogenesis, *in situ* hybridization and immunofluorescence studies demonstrate that *Tbx15* (Supplementary Fig. 6a) and Igf2 were highly and co-expressed in the developing, myosin-positive muscles of embryonic day 14.5 (E14.5) WT mice (Fig. 5d). In agreement with the cellular models, Tbx15 homozygous embryos demonstrated reduced *Igf2* expression in developing muscles (Fig. 5d). This result was confirmed by qPCR of mRNA isolated from dissected limb muscle of E14.5 embryos, which showed 60–65% reductions of *Igf2* mRNA in both the *Tbx15*^*+/−*^ and *Tbx15*^*−/−*^ animals. The levels of Igf2 and Tbx15 were ∼10- and ∼3-fold higher, respectively, at E14.5 than at postnatal day 28 further suggesting a role for Tbx15 during development (Fig. 5e; Supplementary Fig. 6b).

### Igf2 mediates Tbx15 action in skeletal muscle

Since *Igf2* is regulated by Tbx15 levels and since *Igf2* has been implicated to play a role in myogenesis and fibre-type specification^28^, we examined the muscles of *Igf2* knockout mice. As expected, muscle weights of *Igf2* knockout mice were smaller (∼60%) than littermate controls^29^, but there was no obvious change in fibre-type composition. However, like *Tbx15*^*+/−*^ and *Tbx15*^*−/−*^ animals, muscle fibre size in the *Igf2* knockout mice was markedly increased despite the decreased muscle mass (Fig. 5f). Although Igf2 has been thought to be expressed specifically in fast-twitch fibres at E14.5 (ref. 28) and distinct fast- and slow-twitch muscle fibres are found during embryonic development, we found that skeletal muscle of E14.5 embryos expresses fast myosin heavy chain, irrespective of genotype (Supplementary Fig. 6c). However, by day 28, SDH staining revealed clear differences in fibre composition between WT and Tbx15^+*/−*^ muscles (Supplementary Fig. 6d). Taken together, these data suggest that changes in Igf2 levels during embryogenesis do not lead to changes in fibre type, however, loss of Igf2 at this time may lead to alterations in myogenesis and fibre size.

To test whether the change in morphology and size exhibited by *shTbx15* myotubes was due to lack of *Igf2* expression, we treated *shTbx15* and control myotubes with recombinant Igf2 (10 ng ml^−1^) *in vitro* throughout the 4 days of differentiation. Whereas the morphology of control myotubes was not affected by Igf2 treatment, Igf2 treatment partially rescued both the reduced myotube density, as well as the shorter, thicker morphology exhibited by the *shTbx15* myotubes treated with vehicle only (Fig. 5b). Thus, Igf2 appears to be one mediator of Tbx15 action in skeletal muscle with specific effects on regulation of myogenesis and fibre size.

### Tbx15 regulates IGF-2 transcription via an indirect mechanism

Gene regulation and enhancer elements that regulate IGF-2 expression have been extensively studied. The distal enhancer region (CS9) and the proximal promoter region (P3) that are necessary and sufficient for muscle-specific expression of IGF-2 have been previously been defined^30^. Luciferase constructs driven by the P3 promoter element or a combination of the P3 promoter and CS9 enhancer region were transiently transfected into control and shTbx15 C2C12 myoblasts. Transcriptional activity from both the P3 promoter element or the P3 promoter and CS9 enhancer region was reduced by ∼27% (*P*<0.05) in shTbx15 cells compared with control cells in both cases (Supplementary Fig. 6e). To see whether Tbx15 was directly responsible for this effect, we co-transfected the luciferase constructs with different amounts of a Tbx15 expression plasmid into a heterologous cell line, 293FT cells, that do not express Tbx15. In this context, Tbx15 did not transactivate the IGF-2 promoter luciferase constructs (Supplementary Fig. 6f). Taken together, these data suggest that Tbx15 transcriptionally regulates IGF-2, but this effect is most likely indirect.

## Discussion

The ability of muscle to differentiate into different fibre types plays an important role in muscle function and muscle and whole-body metabolism. In the present study, we demonstrate that the mesodermal transcription factor T-box15 is a critical factor controlling the fibre type determination and metabolism of muscle fibres. This is particularly true in the formation of glycolytic fast-twitch fibres. Several other factors have also been demonstrated to increase mass and function of glycolytic muscle fibres and have beneficial effects on glucose homeostasis. Reduction of myostatin signalling by either genetic or pharmacological treatment with soluble activin receptor type IIB leads to an increase in glycolytic muscle mass and improved glucose tolerance^31^,^32^. Similarly, constitutive activation of Akt in skeletal muscle increases glycolytic muscle mass, and results in loss of fat mass and metabolic improvement in mice^33^. A shift from oxidative to glycolytic myofibers by activation of Akt in skeletal muscle also occurs by overexpression of Baf60c and Deptor and this improves glucose metabolism even in the absence of muscle hypertrophy^11^. While all of the above manipulations increase glycolytic fibre density, knockout or knockdown of Tbx15 limits the development of glycolytic muscle fibres.

*Tbx15* is highly and specifically expressed within glycolytic skeletal muscle fibres, and ablation of *Tbx15* leads to marked decrease in number glycolytic fibres and a modest increase in the number of oxidative fibres. The reduction in the ratio of glycolytic to oxidative fibres leads to a muscle with a higher density of oxidative fibres as evidenced by both biochemical and immunofluorescence staining, as well as the slower contraction and relaxation rates, characteristic of oxidative fibres. The loss of glycolytic fibres in *Tbx15*-deficient mice results in a reduction of total muscle mass and total fibre number. Although these reductions do not lead to changes measurable changes in muscle performance either *ex vivo* or *in vivo*, at the whole-body level, the reduction in total muscle mass is associated with decreased spontaneous activity during both the light and dark phases of the diurnal cycle. This ultimately leads to decreased energy expenditure, increased lipid accumulation in adipose tissue and liver, and glucose intolerance. Since Tbx15 is most highly expressed in the skeletal muscle, and other tissues that can have effects on activity, such as the central nervous system, cardiovascular system, lungs and most endocrine organs do not express Tbx15, we believe the defects in skeletal muscle contribute to the reduced activity of the Tbx15-deficient mice.

Although previous cell culture studies have postulated a role for Tbx15 in the development of brown adipose tissues^34^, we found no differences in brown fat formation, morphology or heat production in either the homozygous or heterozygous whole-body *Tbx15*-deficient mice, suggesting a minimal contribution of brown fat to the metabolic phenotypes we observe.

The effect of ablation of *Tbx15* to decrease glycolytic and increase oxidative fibre density occurs with no significant changes in several known regulators of muscle fibre type. Although genes with known higher expression in oxidative fibres, including PGC-1α and RIP140, were tended to be increased in the quadriceps, EDL and soleus muscles on reduction of Tbx15, these results did not reach statistical significance. The increase in these of these genes may reflect the differences in fibre composition on reduction of Tbx15. On the other hand, reduction of *Tbx15* in skeletal muscle and cell lines does result in activation of AMPK, an enzyme known to play a role in muscle fibre determination, skeletal muscle metabolism and regulation of muscle metabolism in response to exercise^35^,^36^. Exactly how loss of Tbx15 activates AMPK signalling remains unknown, but our immunofluorescence and cell fractionation studies clearly demonstrate that a significant fraction of Tbx15 is present in the cytoplasm, in addition to the nucleus, of skeletal muscle cells. Extranuclear localization has been observed for other T-box proteins^37^,^38^. Although we were unable to co-precipitate Tbx15 and AMPK subunits (data not shown), it is possible that Tbx15 and AMPK interact directly or indirectly. Indeed, stable knockdown of *Tbx15* in C2C12 cells recapitulates this effect on AMPK indicating the effect on AMPK occurs in a cell autonomous manner.

Furthermore, activation of the AMPK signalling axis directly represses mTor signalling. Thus, phosphorylation of mTor S2448 and its downstream phosphorylation target p70^S6K1^ T389 were significantly reduced in Tbx15^+*/−*^ muscle in which AMPK activity is high. Although activation of the Akt-mTor signalling axis has been shown to positively regulate fibre size^39^, in the context of reduced Tbx15, fibre size and mTor signalling are not correlated. This suggests that other molecular regulators may be important for fibre size, or that the increase in fibre size might constitute a compensatory hypertrophy triggered by the loss in the number of myofibers.

As noted above, heterozygous and homozygous ablation of Tbx15 leads to dose-dependent decrease in glycolytic fibre number. This loss of glycolytic fibres far exceeds the increase in oxidative fibres leading to an overall decrease in fibre number. Thus, the loss of glycolytic fibres is not simply due to a transformation into oxidative fibres, indicating that Tbx15 is required for normal glycolytic myofiber formation.

At the transcriptional level, one target of Tbx15 is *Igf2*. Analysis of the Igf2 promoter suggests that it may be an indirect target of Tbx15. Consistent with a role as a regulator of myogenesis during embryonic development, the expression of Tbx15 and Igf2 is much higher during embryogeneis than in adult skeletal muscle. Stable knockdown of *Tbx15* in C2C12 cells leads to the development of fewer myotubes, and these myotubes are both shorter and thicker. In Tbx15 homozygous and heterozygous knockout mice, muscle fibres also exhibit increased diameter. *In vitro*, this phenotype is partially rescued by exogenous Igf2 administration indicating that the change in myotube morphology and number is at least partially mediated by Igf2. Furthermore, *Igf2* null animals recapitulate part of the phenotype observed on ablation of *Tbx15* with reduced muscle mass and increased muscle fibre size. However, unlike the ablation of *Tbx15*, knockout of *Igf2* has no effect on the ratio of oxidative to glycolytic muscle fibre. Together, these data indicate that Tbx15 regulates fibre size and number during embryogenesis at least in part via Igf2, but Tbx15 also has a separate role in establishing glycolytic versus oxidative fibre density during postnatal fibre-type formation.

In conclusion, Tbx15 plays a critical role in the formation, contractile properties and metabolism of skeletal muscle through determination of the glycolytic muscle fibre type. As summarized in Fig. 5h, our data indicate that ablation of *Tbx15* results in activation of AMPK signalling and decreased in *Igf2* expression. The increase in AMPK signalling activates oxidative capacity in a cell autonomous manner, whereas the loss of *Igf2* negatively regulates myofiber formation. These two mechanisms contribute to a loss of muscle mass due to a specific loss of glycolytic fibres and an increase in oxidative fibres. This results in a shift of muscle metabolism and function, and ultimately results in a shift of substrates from muscle to fat and liver where they are stored as lipids, leading to increased adiposity and glucose intolerance. Thus, Tbx15 has an important role in the regulation of glycolytic muscle development, and when it fails, this can lead to the development of metabolic syndrome phenotypes, including obesity, hepatosteatosis and glucose intolerance.

## Methods

### Animals and diets

*Tbx15*^*+/−*^ mice on a mixed genetic background^17^ were bred to yield WT, *Tbx15*^*+/−*^ and *Tbx15*^*−/−*^ mice. Mice were allowed *ad libitum* access to water and food containing 22% calories from fat, 23% from protein and 55% from carbohydrates (Mouse Diet 9F 5020; PharmaServ). Male Igf2 knockout mice^29^ and littermate control WT mice on a mixed genetic background were analysed at 6–8 weeks of age. All mice were housed in a mouse facility with a 12-h-light/-dark cycle in a temperature-controlled room. Animal care and study protocols were approved by the Animal Care Committee of Joslin Diabetes Center and were in accordance with the National Institutes of Health guidelines.

### Succinate dehydrogenase staining

Cross-sections of individual skeletal muscles were cut from the mid-belly region of the muscle at 8 μm in a cryostat (−20 °C). After drying for 5 min at room temperature, the sections were incubated for 30 min at 37 °C in a solution (pH 7.6) containing 6.5 mM sodium phosphate monobasic, 43.5 mM sodium phosphate biphasic, 0.6 mM nitroblue tetrazolium (N6876; Sigma) and 50 mM sodium succinate. The sections were then rinsed three times for 30 s each in physiological saline, for 5 min in 15% ethanol and embedded in CC/Mount (Sigma).

### Tissue fractionation

Fractionation of skeletal muscle was performed using a combination of previously described techniques^40^,^41^. Briefly, quadriceps and tibialis anterior muscles were dissected, combined and minced in ice cold PBS with 10 mM EDTA and 0.05% trypsin for 5 min. Tissue piece were washed three times with PBS plus 10 mM EDTA, then homogenized in M1 buffer (0.25 M sucrose, 1 mM EDTA, 10 mM Tris–HCl (pH 7.4), with protease and phosphatase inhibitors (Sigma)) using a Potter-Elvehjem homogenizer. Homogenates were centrifuged at 1,000*g* to remove nuclei and unbroken cells. Nuclei were purified away from unbroken myotubes by filtering through 20 μM cell strainers. The supernatant was then spun at 16,000*g* to pellet mitochondria and plasma membranes (P1) and supernatant (S1). P1 was then resuspended in 0.5 ml of M1 buffer, loaded on 5–25% linear Ficoll gradient and centrifuged for 30 min at 24,000 r.p.m. (SW. 41 rotor). The upper band containing the plasma membrane was removed from the gradient, washed in three volumes of M1 buffer and recentrifuged at 16,000*g* to obtain pure plasma membrane isolates. S1 was then spun in at 48,000 r.p.m. to separate pelleted microsomes and cytosolic proteins.

### Metabolic analysis

For metabolic analysis, 5-month-old *Tbx15*^*+/−*^ and controls were housed individually and evaluated for ambulatory activity using an OPTO-M3 sensor system (Comprehensive Laboratory Animal Monitoring System, CLAMS; Columbus Instruments), which counts beam breaks for 60 s for each mouse, five times per hour during two full light/dark cycles. Indirect calorimetry was measured on the same mice using an open-circuit Oxymax system (Columbus Instruments). After a 48-h acclimation period, exhaust air was sampled for 60 s every 12 min in each cage consecutively for 72 h in the fed state for the determination of O_2_ and CO_2_.

Fed glucose was measured between 0900 hours and 1100 hours in tail vein blood samples (Ascensia Elite). Insulin was measured using rat insulin enzyme-linked immunosorbent assay with mouse standards (Crystal Chem). Intraperitoneal glucose (2 g kg^−1^ weight) and insulin tolerance (1.25 units per kg) tests were performed in unrestrained conscious 5–6-month-old male mice after a 16- and 4-h fast, respectively.

### Measurement of fibre size and type

Tibealis anterior muscle from 8-week-old animals (*n*=4–8 per group) was fixed in 10% formalin and paraffin embedded. Eight-micromitre sections were SDH stained. Five digital images (× 20) from non-overlapping fields were taken from each slide (total 20 fields per group), and fibre areas were calculated using Image J software.

Rectus femoris quadriceps muscle from 8-week-old animals (*n*=4–6 per group) were SDH stained. Whole muscles were photographed by taking overlapping digital images (× 20) and combining the photos using Autostitch software. Fibres were counted using Image J software.

### Gene expression

Analysis of gene expression was conducted using qPCR. Total RNA was extracted using an RNeasy minikit (QIAGEN), and 3 μg was reverse transcribed in 100 μl using a High Capacity cDNA Reverse Transcription Kit (Applied Biosystems). Five microlitre of diluted complementary DNA from the reverse transcription reaction (1/10) was amplified with specific primers (300 nM each) in a 10-μl PCR reaction with a SYBR green PCR master mix (Applied Biosystems). Analysis of gene expression was carried out in an ABI Prism 7900 sequence detector with an initial denaturation at 95 °C for 10 min, followed by 40 PCR cycles, each consisting of 95 °C for 15 s and 60 °C for 1 min. SYBR green fluorescence emission was monitored after each cycle. For each gene, mRNA expression was calculated relative to TBP. Amplification of specific transcripts was confirmed by melting curve profiles at the end of each PCR. Primer sequences are listed in Supplementary Table 1.

### Western blot

Proteins were extracted from cells in RIPA buffer with 0.1% SDS. Thirty microgram of protein was subjected to SDS–polyacrylamide gel electrophoresis and transferred to polyvinylidine fluoride membranes. Uncropped images of gels of key western blot experiments are shown in Supplementary Fig. 7. Primary antibodies listed in Supplementary Table 2.

### Immunofluorescence

Immunofluorescence was performed on frozen sections. Specific signals were detected with Alexa Fluor 564-, Alexa Fluor 488- and Alexa Fluor 405-conjugated secondary antibodies (1:500). Alexa Fluor 546 phalloidin (Life technologies) was used according to manufacturer’s instructions. Primary antibodies are listed in Supplementary Table 2.

### Isometric contraction measurements

The EDL was removed by dissection and kept in oxygenated Krebs–Henseleit solution (in mM): 120 NaCl, 3.3 KCl, 1.2 MgSO_4_, 1.2 KH_2_PO_4_, 1.3 CaCl_2_, 25 NaHCO_3_, aerated with 5% CO_2_/95% O_2_. A fibre bundle of EDL was prepared in a Petri dish with spring scissors under a Wild M8 microscope (Leica). To measure isometric contractions, the fibre bundle was transferred into a chamber that was perfused with oxygenated 25 mM HCO_3_^−^/5% CO_2_ buffered Krebs–Henseleit solution^42^. One end of the fibre bundle was fixed; the other was connected to a force transducer (SensoNor, Friedberg, Germany). The length of the fibre bundle was adjusted to give maximal isometric twitch tension. The fibres were directly stimulated by platinum wires at 25 °C (stimulator S 48, Grass Instrument Division, W. Warwick, RI, USA). Single twitches were triggered by pulses of 1-ms duration and supramaximal voltage and tetani by pulses of 1-ms duration at 100 Hz for 400 ms.

Recordings of single twitches were analysed for peak force, TTP and 50 and 75% relaxation times (t50 and t75%), respectively. Tetani were analysed for maximum tetanic force, t50 and t75%. The TTP is defined as the time interval between the time at which the trace deviates from baseline and the time at which peak force is achieved. t50 and t75% are defined as the time interval between the peak value and the time at which the signal has decayed to 50 and 75% of peak force.

### *Ex vivo* contraction

Muscle force production was determined as previously described^43^,^44^,^45^,^46^ with some modifications. Mice were anaesthetized (90 mg kg^−1^ pentobarbital intraperitoneal), killed by cervical dislocation and EDL muscles rapidly excised. Muscle length was measured with a micrometre, and muscles attached to a tissue support with stimulating electrodes (Harvard Apparatus, Holliston, MA, USA). Muscles were bathed in Krebs-Ringer Bicarbonate buffer containing (in mM): 117 NaCl, 4.7 KCl, 2.5 CaCl_2_·2H_2_O, 1.2 KH_2_PO_4_, 1.2 MgSO_4_·7H_2_O, 24.6 NaHCO_3_, 5.6 glucose at 30 °C, pH 7.4, and continuously gassed with 95% O_2_/5% CO_2_. Optimal muscle length (*L*_O_) was determined for each muscle by increasing the resting tension to 2.40 g and decreasing resting tension by 0.4 g increments and applying a single electrical pulse generated by a Grass stimulator (Harvard Apparatus, Holliston, MA, USA; parameters: pulse rate=1 pulse per second; duration=1 ms; volts=100 V). The tension (*L*_O_) at which peak force was achieved was the tension applied to perform to the following contraction protocols. Force production was monitored using isometric force transducers (Kent Scientific, Litchfield, CT, USA), the converted digital signal was captured by a data acquisition system (iWorx114, CB Sciences, Dover, NH, USA), and assessed with analysis software (Labscribe, CB Sciences). Specific force was calculated by normalizing force measurements to total muscle CSA. Total CSA was determined by dividing muscle weight by the product of the muscle length and the average density of mammalian skeletal muscle (1.06 mg mm^−3^). Maximal twitch force was assessed by averaging four muscle twitches, maximally stimulated using the following electrical pulse parameters: (pulse rate=1 pulse per second; duration=1 ms; volts=100 V). Maximal tetanic force was assessed by averaging four tetanic contractions, maximally stimulated using the following electrical pulse parameters: (pulse rate=130 pulse per second; duration=1 ms; volts=100 V). Muscle fatigue rate was assessed by rate of decay of maximal twitch force during 40 repeated stimulations.

### Test of muscle performance

Six-week-old Tbx15^+*/−*^ and male littermate control mice were randomly assigned to housing in individual cages with or without running wheels (Nalgene, Rochester, NY, USA) for a total of 3 weeks. Completed wheel revolutions and time spent running were monitored daily. On day 21, all mice were removed from their cages being killed by cervical dislocation.

Eight-week-old Tbx15^+*/−*^ and male littermate control mice were familiarized with the treadmill (Quinton model 42) by running 5–10 min 2 days before the onset of experimentation. Animals were then subjected to an exhaustive ‘ramp’ protocol, as previously described^47^. Briefly, WT and Tbx15^+*/−*^ male mice at 8 week of age were subjected to a graded ramp treadmill running protocol. The treadmill speed began at 0.2 miles per hour and increased 0.2 miles per hour every 3 min until the speed reached 0.8 miles per hour. The incline was increased by 2.5% every 3 min, to a maximum of 20%. The speed and incline were then kept constant (0.8 mile per hour and 20%) until the mice reached exhaustion. Following acclimation, muscle strength of 8-week-old Tbx15^+*/−*^ and control mice was measured using an automated Grip Strength Meter (Columbus Instruments).

### Reporter assays

For regulation of IGF-2 constructs: shTbx15 and control C2C12 cells or 293FT cells at 70% confluence in DMEM with 10% fetal bovine serum grown to were co-transfected with 1 μg of a previously described of IGF-2 promoter constructs^30^, pBABE-Tbx15 or BABE-Empty and pRenilla-TK (Promega), using Superfect transfection reagent (Quiagen). All transfections were harvested 24 h later and luciferase activity was measured using a Dual Renilla Luciferase II Assay Kit, and normalized to Renilla luciferase measurements (Promega).

### Retroviral and lentiviral infection

Short hairpin RNA knockdown of *Tbx15* was achieved in mycoplasma-free (as tested with Plasmotest (InvivoGen)) C2C12 cells (ATCC) by lentiviral infection. Plates (10 cm) of 70% confluent 293FT cells were transiently transfected with 1 μg of lentiviral *shTbx15* in pLKO.1 (Catalog #:TRCN0000084362) and the viral packaging vectors SV-E-MLV-env and SV-E-MLV using Superfect (Qiagen) according to the manufacturer’s instructions. Forty-eight hour after transfection, virus-containing medium was collected and passed through a 0.45-μm-syringe filter. Filter-sterilized Polybrene (hexadimethrine bromide, 8 μg ml^−1^) was added to the virus-loaded medium, and the medium was applied to proliferating cells. Forty-eight hour after infection, cells were treated with trypsin and replated in a medium supplemented with puromycin (Invitrogen) as a selection antibiotic.

*Tbx15* was stably overexpressed in C3H10T1/2 cells by retroviral infection. Phoenix cells were grown to 70% confluency in 10-cm plates and were transiently transfected with 1 μg of retroviral expression vectors pBABE-Empty-puro or pBABE-*Tbx15-*puro using Superfect (Qiagen) according to the manufacturer’s instructions. At 48 h after transfection, virus-containing medium was collected and passed through a 0.45-μm pore-size syringe filter. Filter-sterilized Polybrene (8 μg ml^−1^ hexadimethrine bromide) was added to the virus-loaded medium, and the medium was then applied to proliferating (20% confluent) cells. At 48 h after infection, cells were treated with trypsin and replated in a medium supplemented with zeocin (Invitrogen) as a selection antibiotic.

Cells were grown in DMEM containing high glucose (4,500 mg l^−1^) with 10% fetal bovine serum (Gemini). Differentiation was induced by treating cells with 2% heat-inactivated horse serum (Sigma) in DMEM containing high glucose (4,500 mg l^−1^) for 4 days.

### Microarray analysis of gene expression

RNA was extracted and biotin-labelled complementary RNA (cRNA) was prepared from three independent transfections of the four stable C2C12 myoblast cell lines described above: *shTbx15*, shGFP, pBABE-Empty-puro and pBABE-*Tbx15-*puro. Complementary RNA was hybridized to Affymetrix M430 2.0 arrays, and microarray analysis was performed on globally scaled data (MAS 5.0). Gene set enrichment analysis and ingenuity pathway analysis were used to determine significantly regulated pathways and gene networks.

### Oil Red O staining

Liver samples from five 5-month-old male *Tbx15*^*+/−*^ and littermate male control mice were embedded in OCT and sectioned 9–10-μm thick. Oil Red O stock solution (0.5 g of Oil Red O (Sigma) in 100 ml of isopropanol) was diluted with water (60:40 (vol/vol)), followed by filtration. After staining, samples were washed several times in water. Liver sections were lightly counterstained with haematoxylin. Pictures are taken at × 20.

### XF24 oxygen consumption assay and oxygen consumption rate

Oxygen consumption was measured using the XF24 Extracellular Flux Analyser from Seahorse Bioscience. For this, 2 h before the analysis, control, sh*Tbx15* and Tbx15 overexpressing C2C12 myoblasts on 24-well XF24 V28 cell culture microplate (Seahorse Bioscience) were pretreated compound C. One hour before the experiment, cells were washed and incubated in 630 μl of non-buffered (without sodium carbonate) DMEM (4.5 g l^−1^ glucose) pH 7.4 at 37 °C in a non-CO_2_ incubator. Five replicates per cell type were included in the experiment, and four wells evenly distributed within the plate were used for correction of temperature variations. During the time course of the experiment, oxygen concentration was measured over time periods of 2 min at 6-min intervals, consisting of a 2 min of mixing period and a 4 min waiting period. OCR over the 2 min measurement period was calculated using the Fixed Delta technique for determining the slope.

### Study design and statistics

Studies were conducted in a non-blinded fashion. For all studies, samples were not randomized, and sample size (range from *n*=3 to *n*=14) are explicitly stated in the figure legends. Normal distribution was assessed by the Jarque–Bera test (*P*>0.05). All differences were analysed by analysis of variance, or Student’s *t*-test if normally distributed. The Mann–Whitney *U*-test was used for data that was not normally distributed. Results were considered significant if *P*<0.05.

## Additional information

**Accession code**: Microarray data have been deposited into the NCBI GEO data repository under accession code GSE70489.

**How to cite this article:** Lee, K. Y. *et al.* *Tbx15* controls skeletal muscle fibre-type determination and muscle metabolism. *Nat. Commun.* 6:8054 doi: 10.1038/ncomms9054 (2015).

## Supplementary Material

Supplementary Information Supplementary Figures 1-7, Supplementary Tables 1-4
