# Supplementary material for: Tbx15 controls skeletal muscle fibre-type determination and muscle metabolism
Source: Nat Commun. 2015 Aug 24;6:8054. doi: 10.1038/ncomms9054 (PMC4552045; doi:10.1038/ncomms9054)
Supplement: Supplementary Information — Supplementary Figures 1-7, Supplementary Tables 1-4 [file ncomms9054-s1.pdf]

Supplementary Figure 1

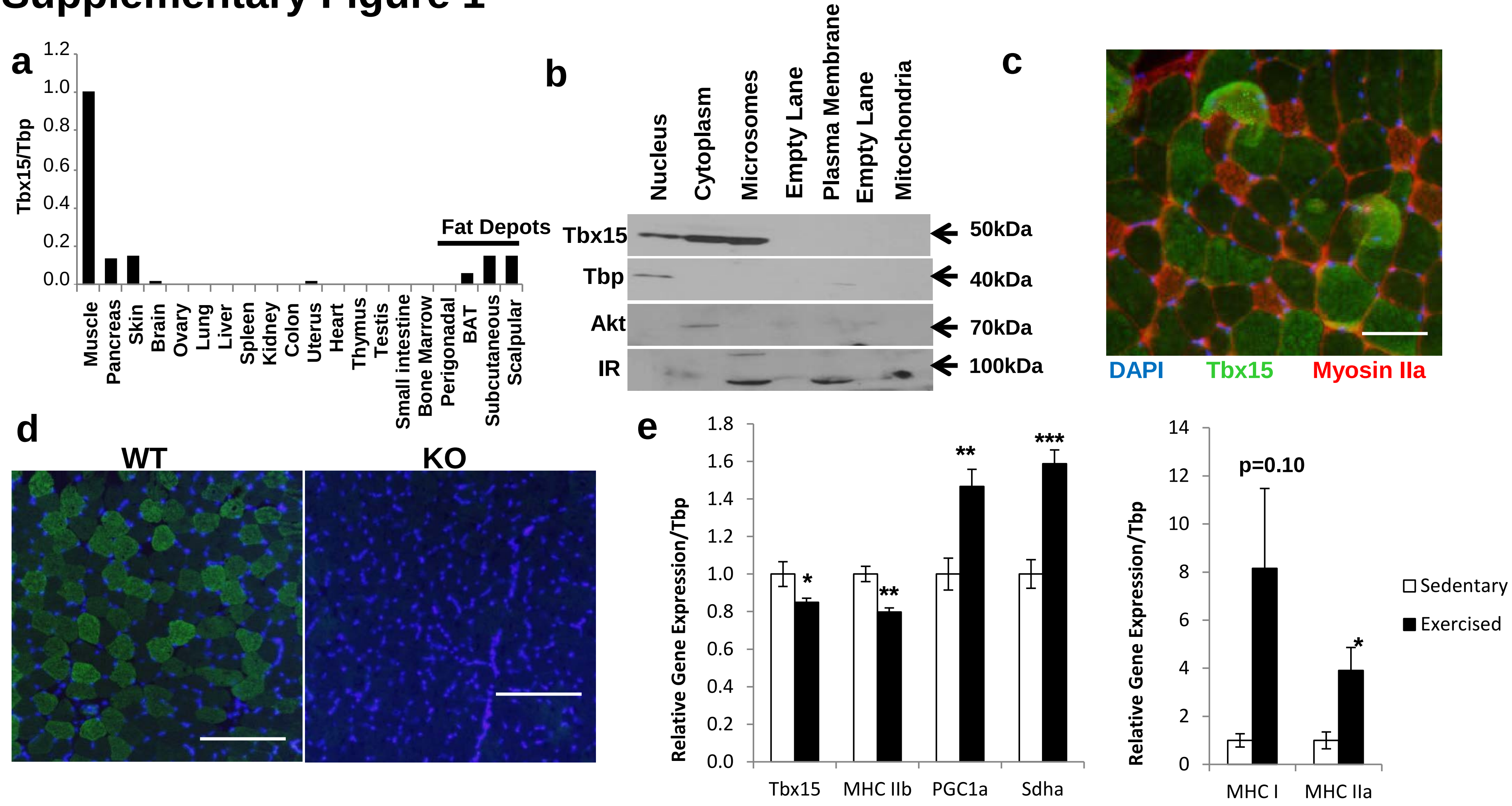

Supplementary Figure 1. *Tbx15* is up-regulated during C2C12 differentiation.
a) Expression level of *Tbx15* mRNA was compared using quantitative real-time PCR (qPCR) of RNA isolated from tissues from of 8 week old male and female (ovary and uterus) C57BL/6 mice. Data are shown as mean ± SEM of four samples.
b) Western blot for Tbx15 from of fractionated muscle tissue from 8 week old mice. Tata binding protein (Tbp), Akt, and insulin receptor (IR) are run as controls.
c) Double immunofluorescence for Tbx15 (green) and myosin IIa (red) on tibialis anterior muscle of 8 weeks old mice. The photograph was taken at 10x magnification. Scale bar =50 µM.
d) Immunofluorescence for Tbx15 (green) on tibialis anterior muscle of WT and $Tbx15^{-/-}$ at 8 weeks old mice. The photograph was taken at 10x magnification. Scale bar =100 µM.
e) Expression level of *Tbx15* mRNA, myosin heavy chains, and markers of oxidative metabolism was compared using quantitative real-time PCR (qPCR) of RNA isolated from triceps of from of male C57BL/6 mice given free access to wheel cages for three weeks to those house in standard cages. Data are shown as mean ± SEM of six samples.

Supplementary Figure 2a-c

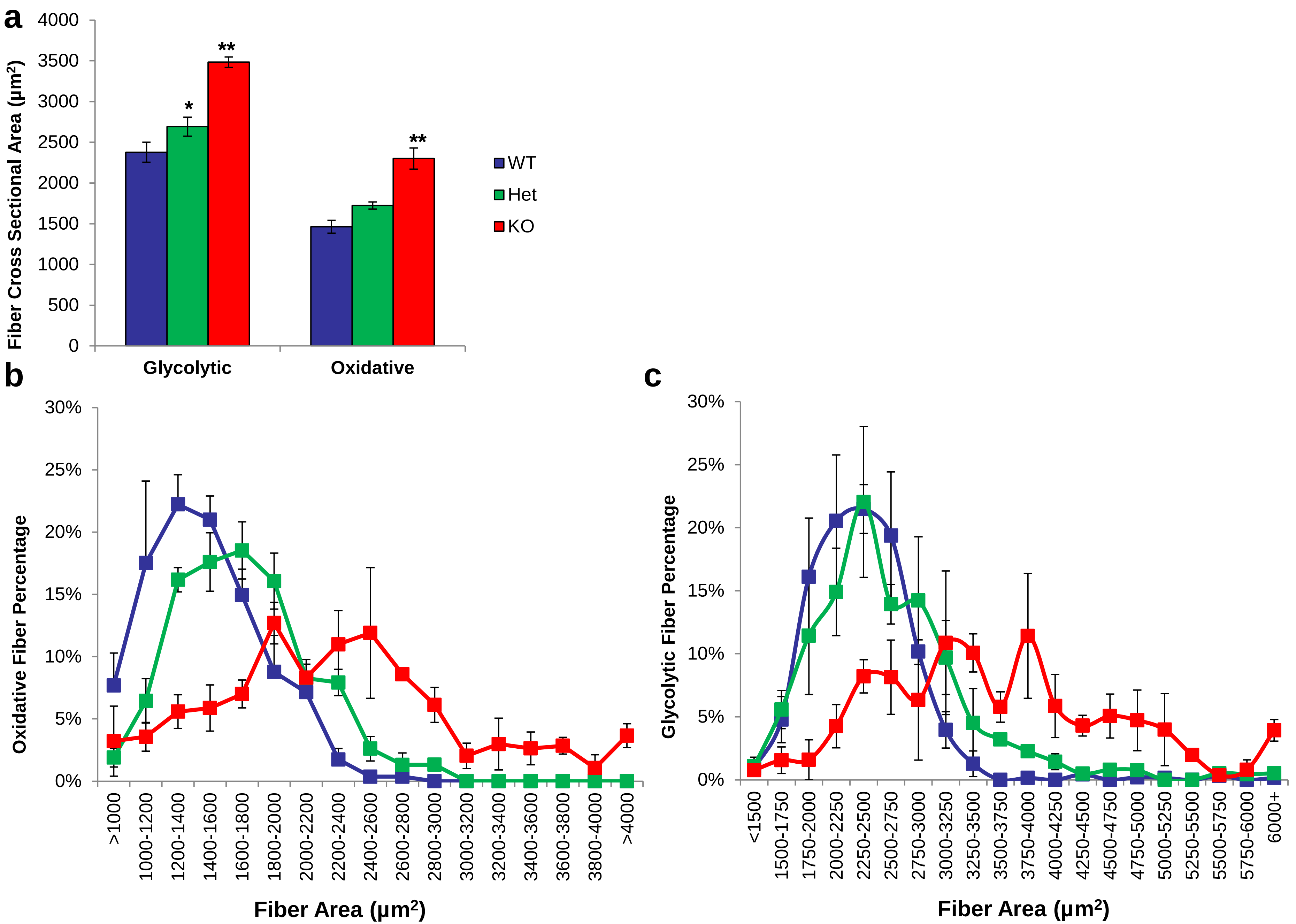

Supplemental Figure 2d-g

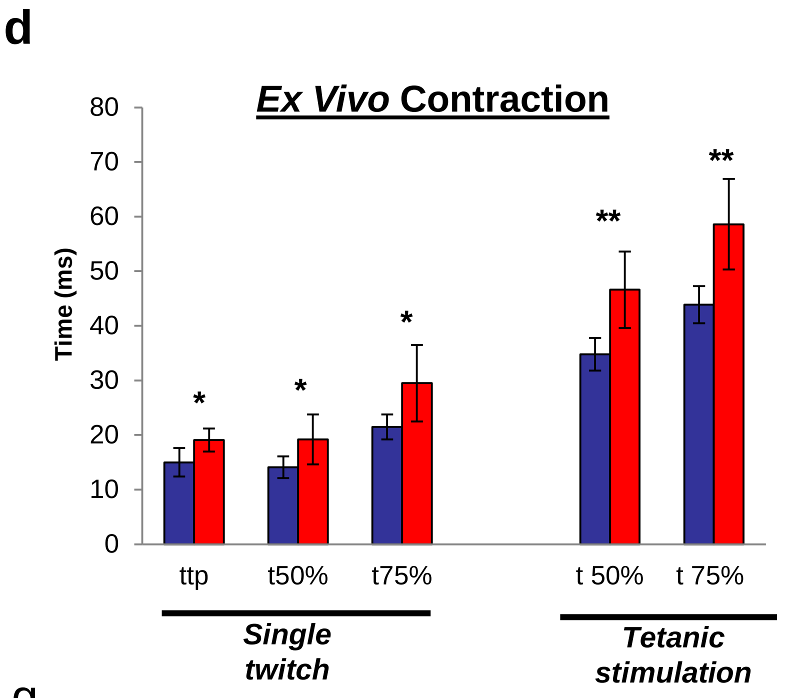

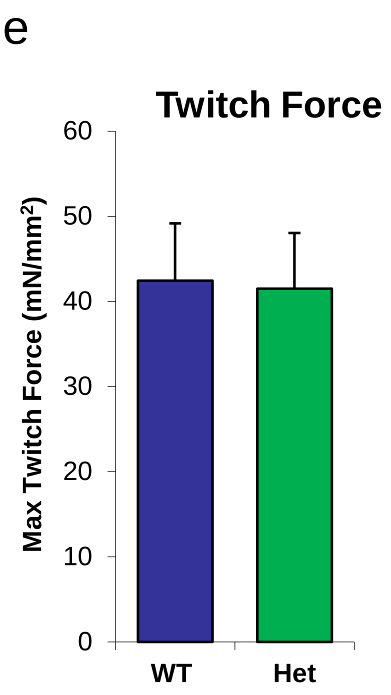

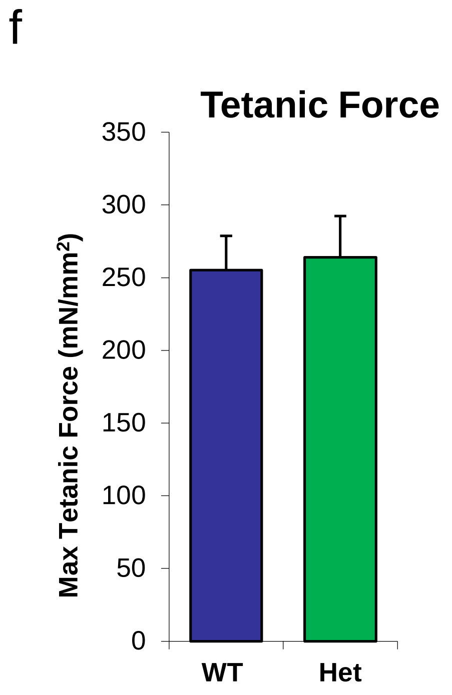

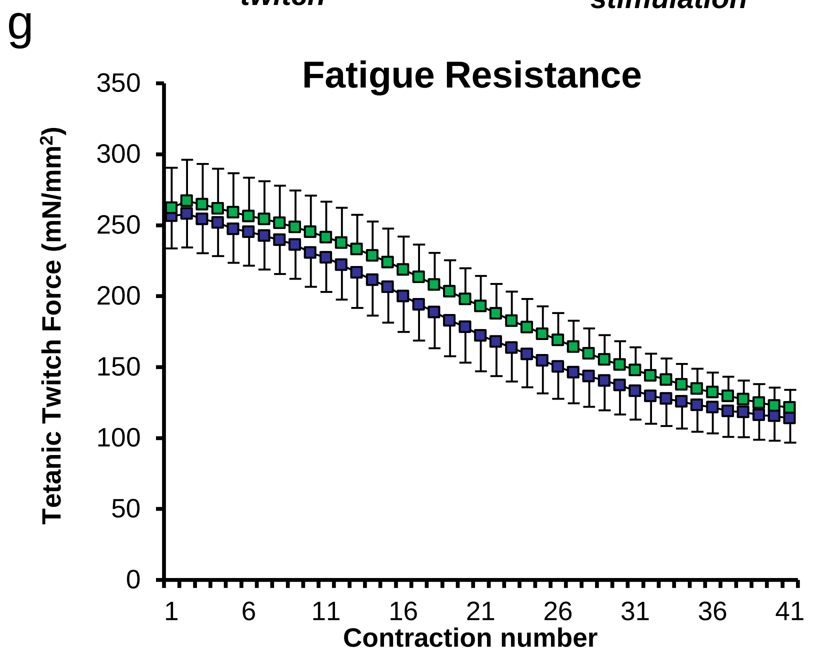

Supplementary Figure 2h-j

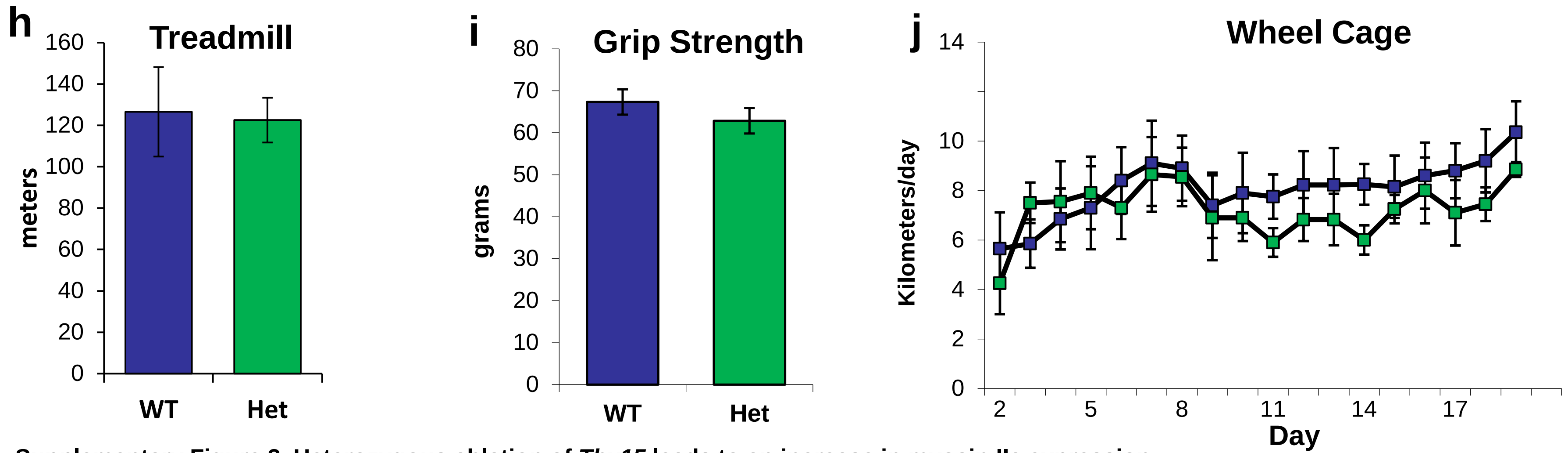

Supplementary Figure 2. Heterozygous ablation of *Tbx15* leads to an increase in myosin IIa expression.

a) Cross sectional area of oxidative and glycolytic skeletal muscle fibers from quadriceps muscles of male WT, *Tbx15*$^{+/-}$ and *Tbx15*$^{-/-}$ mice at 6-8 weeks. Five digital images (20x) from non-overlapping fields were taken from mouse muscle (total 15-20 fields per group), and oxidative and glycolytic muscle fibers separately analyzed. Data are shown as mean ± SEM of 3-4 animals per group. Asterisks indicate a significant differences in all panels (* $p<0.05$; ** $p<0.01$ by Mann-Whitney U test)

b) Distribution of oxidative fiber diameter from WT, *Tbx15*$^{+/-}$ and *Tbx15*$^{-/-}$ mice from Supplementary Figure 2a. Fiber type distribution is broader in *Tbx15*$^{+/-}$ and *Tbx15*$^{-/-}$ mice ($p<0.05$, and $p<0.001$ by Mann-Whitney U test compared to WT for oxidative fibers).

c) Distribution of glycolytic fiber diameter from WT, *Tbx15*$^{+/-}$ and *Tbx15*$^{-/-}$ mice from Supplementary Figure 2a. Fiber type distribution is broader in *Tbx15*$^{+/-}$ and *Tbx15*$^{-/-}$ mice ($p<0.05$, and $p<0.01$ by Mann-Whitney U test compared to WT for glycolytic fibers).

d) Quantitation of single twitch contraction and tetanic stimulation *ex vivo* normalized to muscle cross sectional area. The ttp (time to peak) is defined as the time interval between the time at which the trace deviates from baseline and the time at which peak force is achieved. T50% and t75% are defined as the time interval between the peak value and the time at which the signal has decayed to 50% and 75% of peak force. Data are shown as mean ± SEM of 8-10 muscle fibers per group.

e) Quantitation of maximal twitch force of single twitch contraction of EDL fiber bundles *ex vivo* from WT and *Tbx15*$^{+/-}$ mice at 3-4 months of age. Data are shown as mean ± SEM of 7-9 muscle fibers per group and are normalized to cross-sectional area of fibers.

f) Quantitation of maximal tetanic force of single twitch contraction of EDL fiber bundles *ex vivo* from WT and *Tbx15*$^{+/-}$ mice at 3-4 months of age. Data are shown as mean ± SEM of 7-9 muscle fibers per group and are normalized to cross-sectional area of fibers.

g) *Ex vivo* measurements of tetanic twitch force in EDL fiber bundles from WT and *Tbx15*$^{+/-}$ mice at 3-4 months of age in response to repeated contraction. Data are shown as mean ± SEM of 7-9 muscle fibers per group and are normalized to cross-sectional area of fibers.

h) Total distance run of 8 week old Tbx15$^{+/-}$ and control males on the treadmill in a forced running paradigm. Data are shown as mean ± SEM of 6-8 animals/group.

i) Grip strength measured in 8 week old Tbx15$^{+/-}$ and control males. Data are shown as mean ± SEM of 12-14 animals/group.

j) Daily distance run of 6-9 week old Tbx15$^{+/-}$ and control males given free access to wheel cages over three weeks. Data are shown as mean ± SEM of 6 animals/group.

Supplementary Figure 3

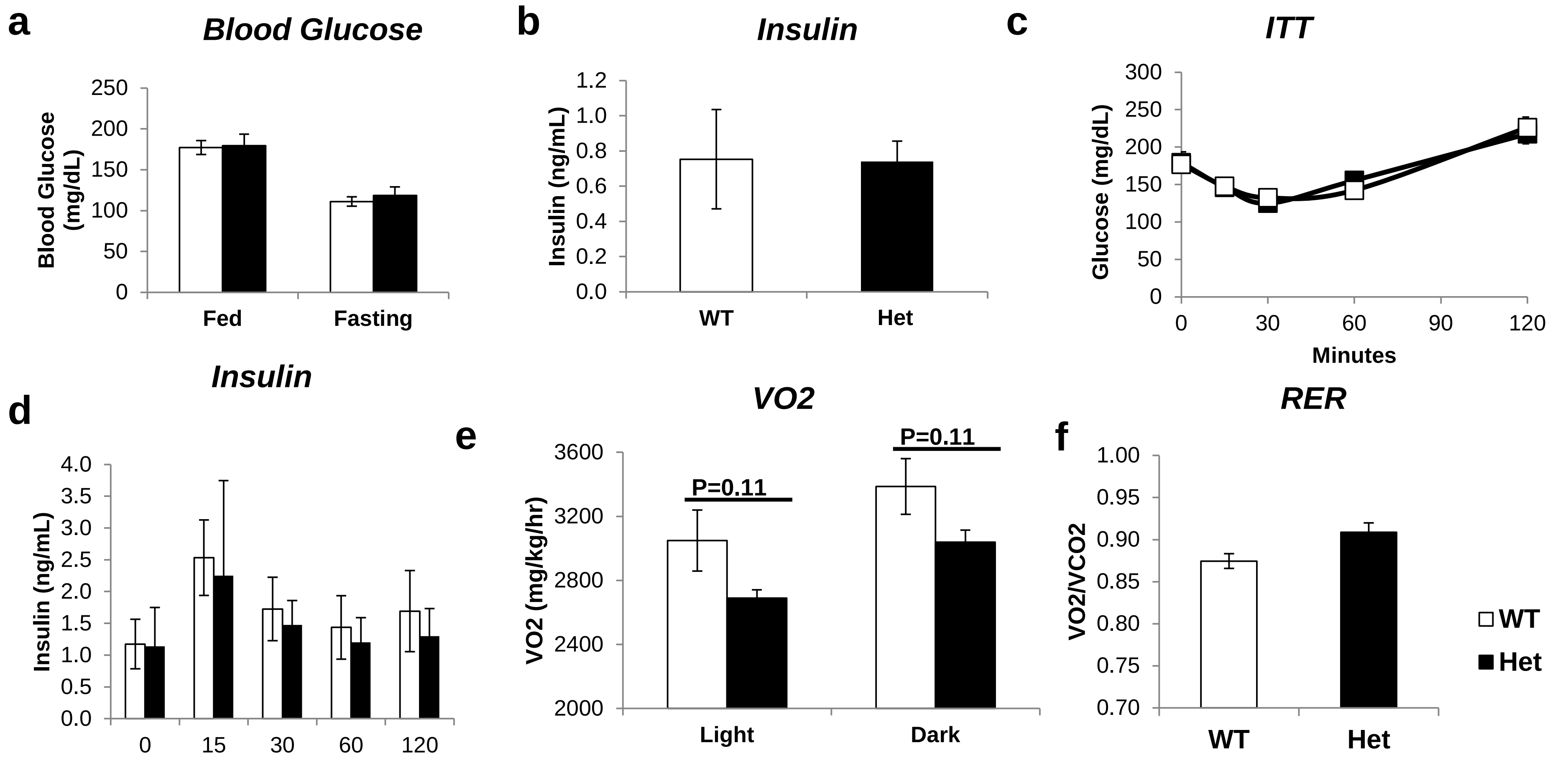

Supplementary Figure 3. Heterozygous ablation of *Tbx15* leads to reduced energy expenditure but does not alter blood glucose levels, insulin levels, or insulin tolerance.

a) Blood glucose measurement of 5 month old *Tbx15*$^{+/-}$ and control males in the random fed state and after overnight fast. Data are the means ± SEMs of 10-12 animals/group.

b) Serum insulin levels from random-fed 5 month-old *Tbx15*$^{+/-}$ and control males. Data are the mean s± SEMs of 6-7 animals per group.

c) Insulin tolerance tests of in 6 month old *Tbx15*$^{+/-}$ and control males. Data are the means ± SEMs of 10-12 animals per group. (* $p<0.05$ for all panels by Student's t-test)

d) Serum insulin levels as measured during glucose tolerance testing of 5 month-old *Tbx15*$^{+/-}$ and control males. Data are shown as mean ± SEM of 6 animals/group.

e) Oxygen consumption during the light and dark phase of the diurnal cycle, as measured by indirect calorimetry in 6 month old *Tbx15*$^{+/-}$ and control males. Data are the means ± SEMs of 6 animals per group.

f) Respiratory exchange ratio (RER) in 6 month old *Tbx15*$^{+/-}$ and control males during the light phase of the diurnal cycle as measured by indirect calorimetry. Data are the means ± SEMs of 6 animals per group.

Supplementary Figure 4

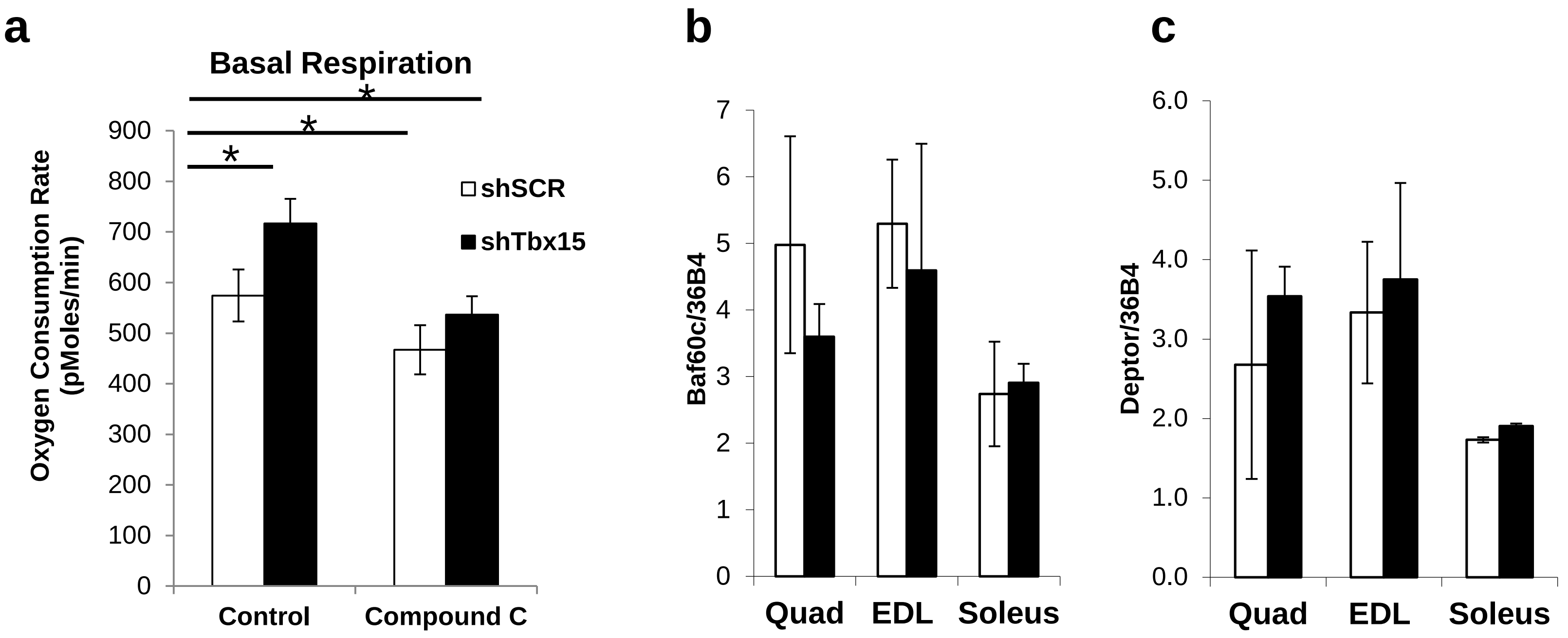

Supplementary Figure 4. Inhibition of AmpK reduces basal respiration in both *shTbx15*and control C2C12 myoblasts.
a) Basal respiration of *shTbx15*and control C2C12 myoblasts pretreated with Compound C (20 µM) for 2 hours. Values are means ± SEM of 5 replicates. The whole experiment was repeated three times. (*p<0.05 for all panels by Student's t-test)
b-c) qPCR analysis for *Baf60c,* and *Deptor* mRNA of RNA isolated from the tibialis anterior of male wild-type (WT) and *Tbx15*$^{+/-}$ at 6-8 weeks of age. Data are shown as mean ± SEM of 6 animals per group.

Supplementary Figure 5

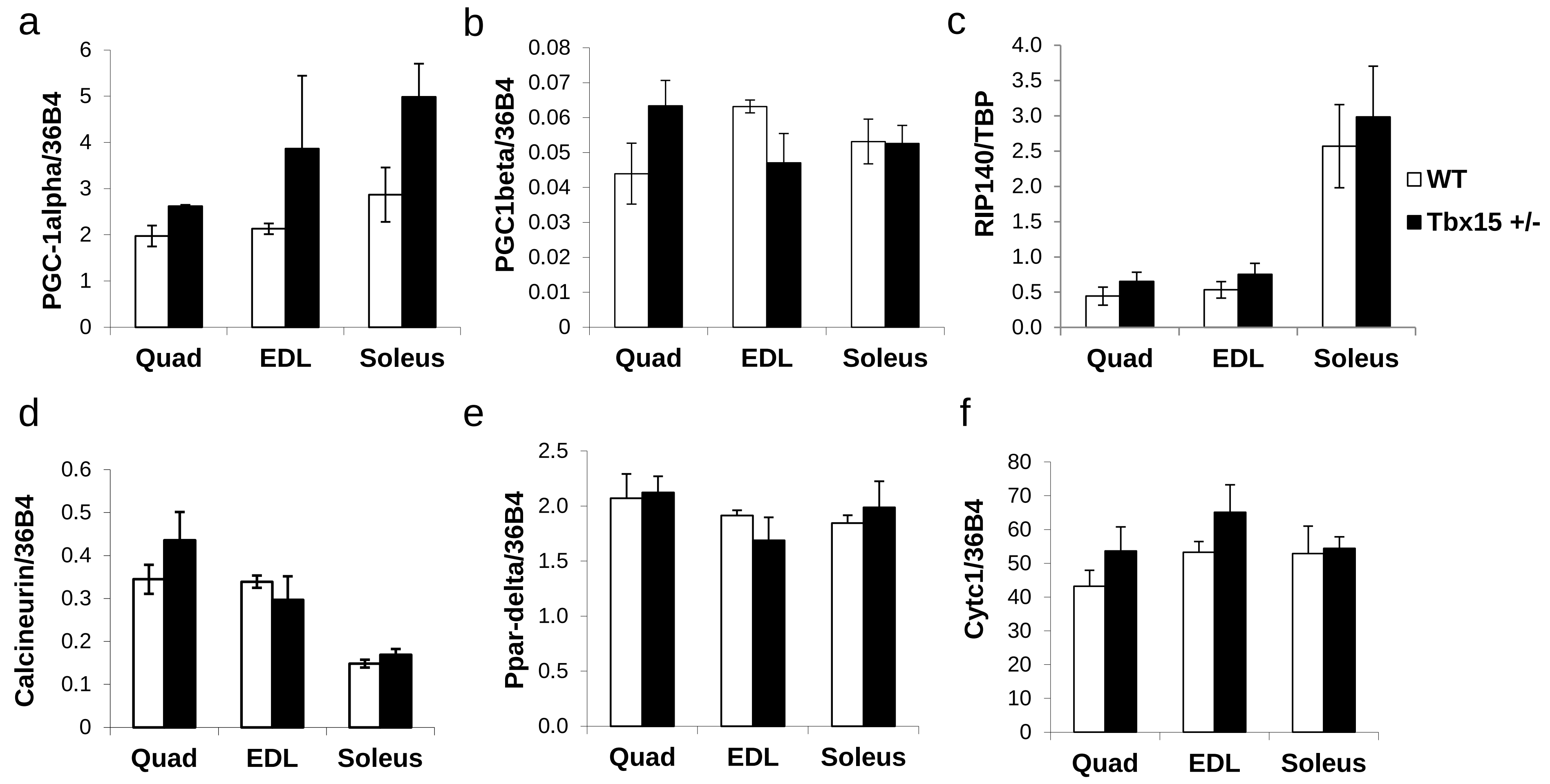

Supplementary Figure 5. *Tbx15* ablation does not lead to changes in known mediators of muscle fiber-type.
a-f) qPCR analysis of mRNA of known markers of muscle fiber-type of RNA isolated from the tibialis anterior of male wild-type (WT) and *Tbx15*$^{+/-}$ at 6-8 weeks of age. Data are shown as mean ± SEM of 6 animals per group.

Supplementary Figure 6a-d

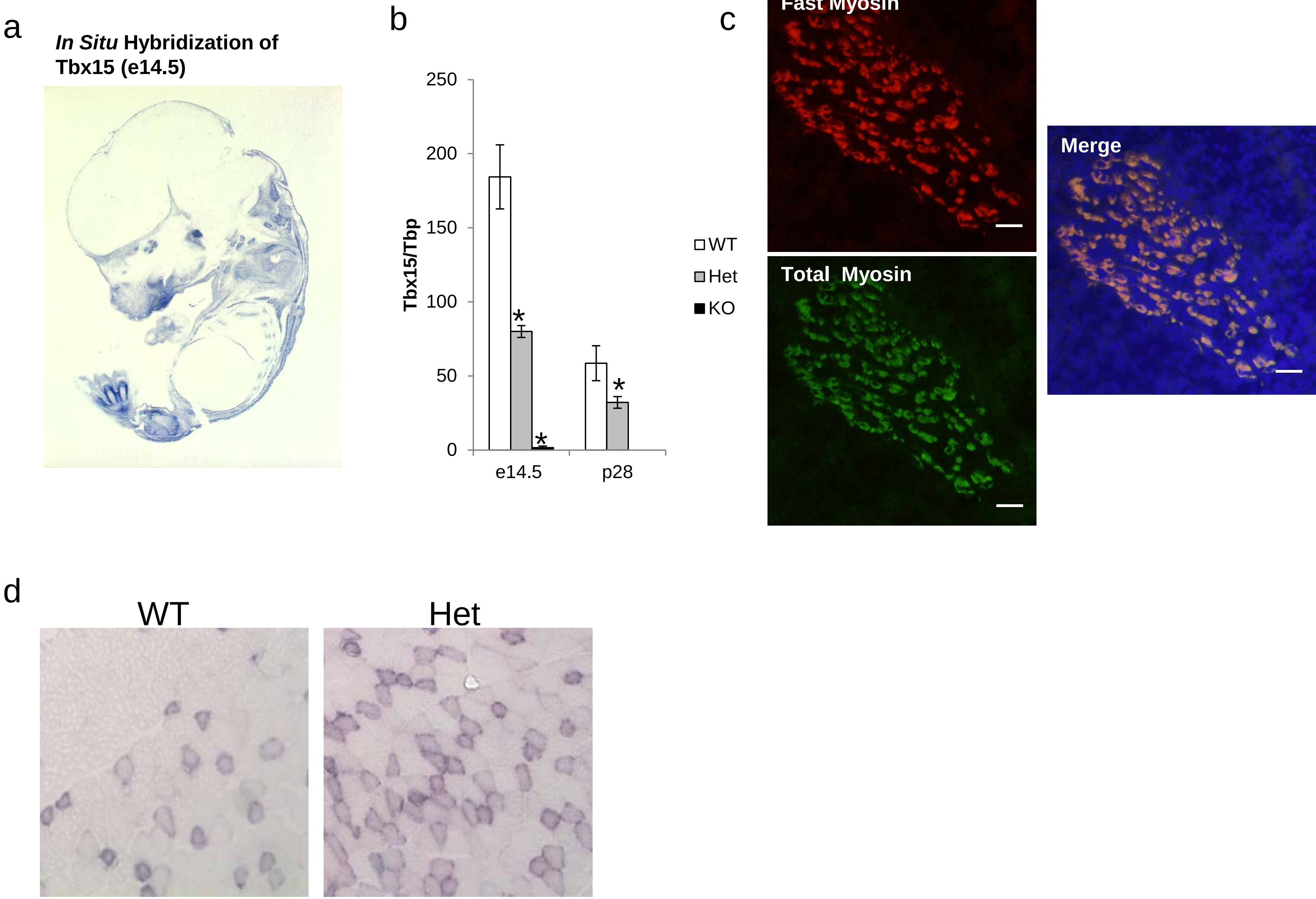

Supplementary Figure 6e-f

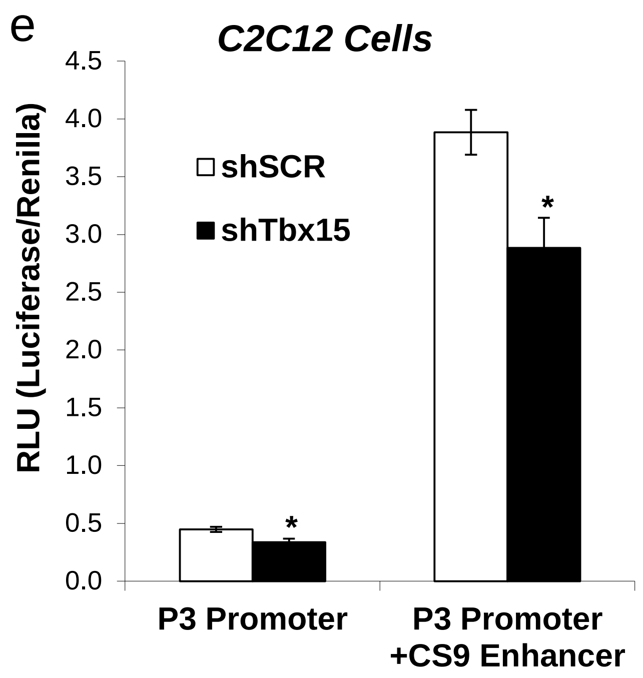

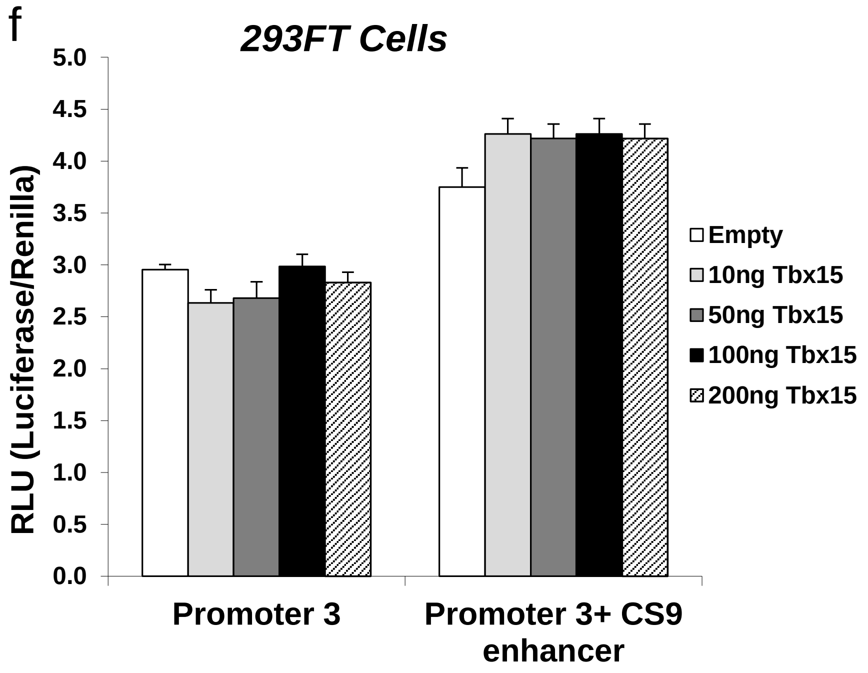

Supplementary Figure 6. Tbx15 is expressed in developing muscles.
a) In situ hybridization for Tbx15 expression on sagittal sections of E14.5 mouse embryos.
b) Expression level of Tbx15mRNA was compared by qPCR in dissected limb muscle from WT, Tbx15+/- and Tbx15-/- e14.5 embryos and from tibialis anterior skeletal muscle from at WT and Tbx15-/- male mice at p28. Values are means ± SEM of 4-6 animals per group.
c) Immunofluorescence staining for fast myosin isoforms (red) and total myosin isoforms (green) and merged picture from developing muscles in the limb buds of wild-type (WT) E14.5 embryos. Pictures are taken at 10x magnification. Scale bar = 100 µM.
d) Succinate dehydrogenase (SDH) of tibealis anterior from wild-type (WT) and Tbx15+/- male mice at d28. SDH pictures are taken at 20x.
e) Luciferase activity of shTbx15 and control C2C12 cells transfected with the IGF-2 muscle specific promoter P3, or P3 with the CS9 distal enhancer. Cells were harvested after 24 hrs and luciferase readings were normalized to Renilla luciferase. Data shown as mean ± SEM of three replicates and was repeated twice. (*$p<0.05$ for all panels by Student's t-test)
f) Luciferase activity of 293FT cells transfected with the IGF-2 muscle specific promoter P3, or P3 with the CS9 distal enhancer. Cells were co-transfected with Tbx15 expression plasmid and harvested after 24 hrs and luciferase readings were normalized to Renilla luciferase. Data shown as mean ± SEM of three replicates and was repeated twice.

Supplementary Figure 7. Uncropped Western Blots of Key Experiments

Figure 1b. C2C12 Differentiation

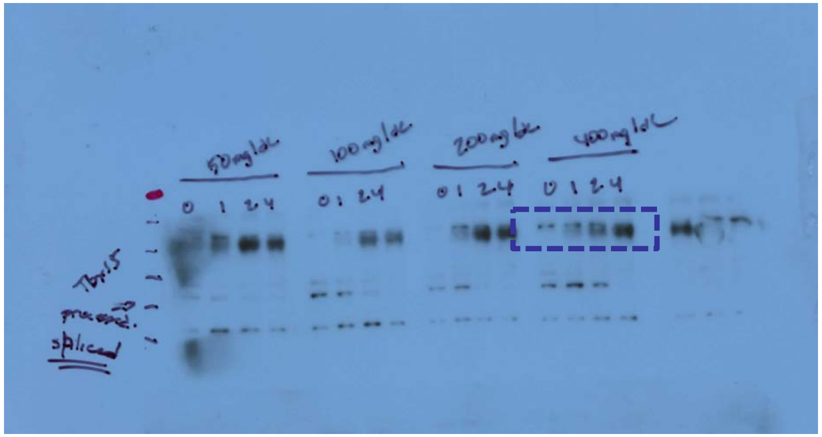

Figure 2a. Tbx15 Levels in Tbx15$^{+/-}$ and Tbx15$^{-/-}$ Skeletal Muscle

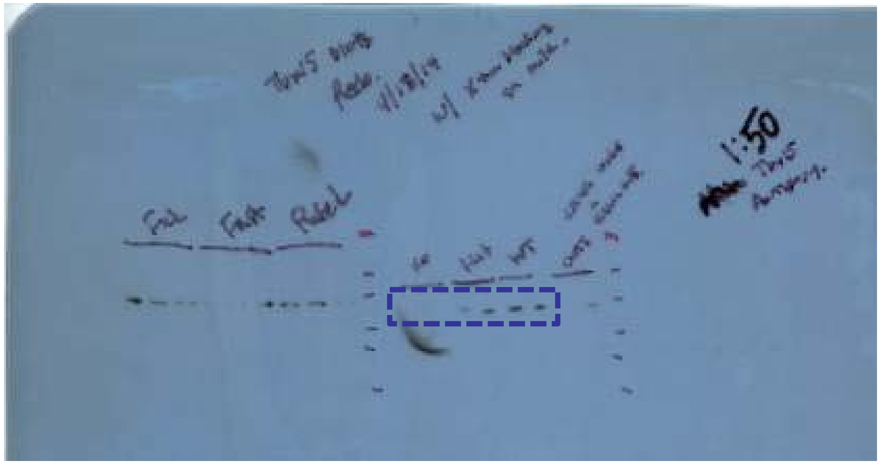

Figure 3c. AMPK Pathway Activation in Tbx15$^{+/-}$ Skeletal Muscle

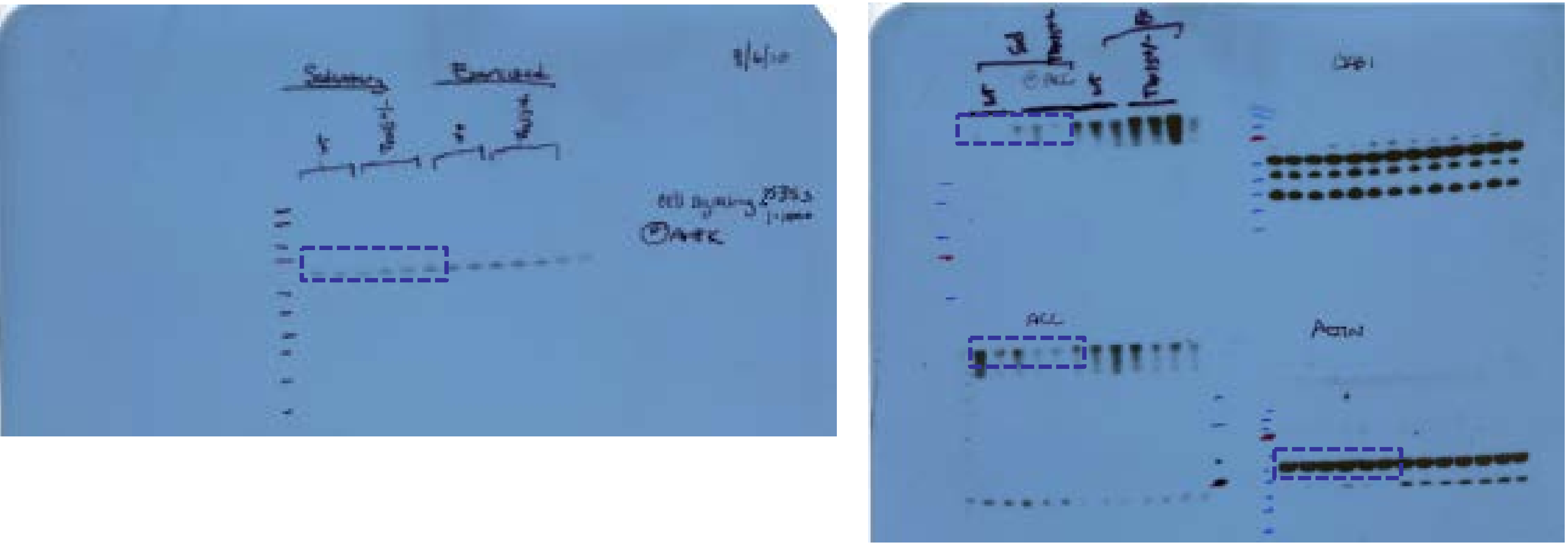

Figure 4e. Inhibition of mTOR and and pS6K1 Signaling in Tbx15$^{+/-}$ Skeletal Muscle

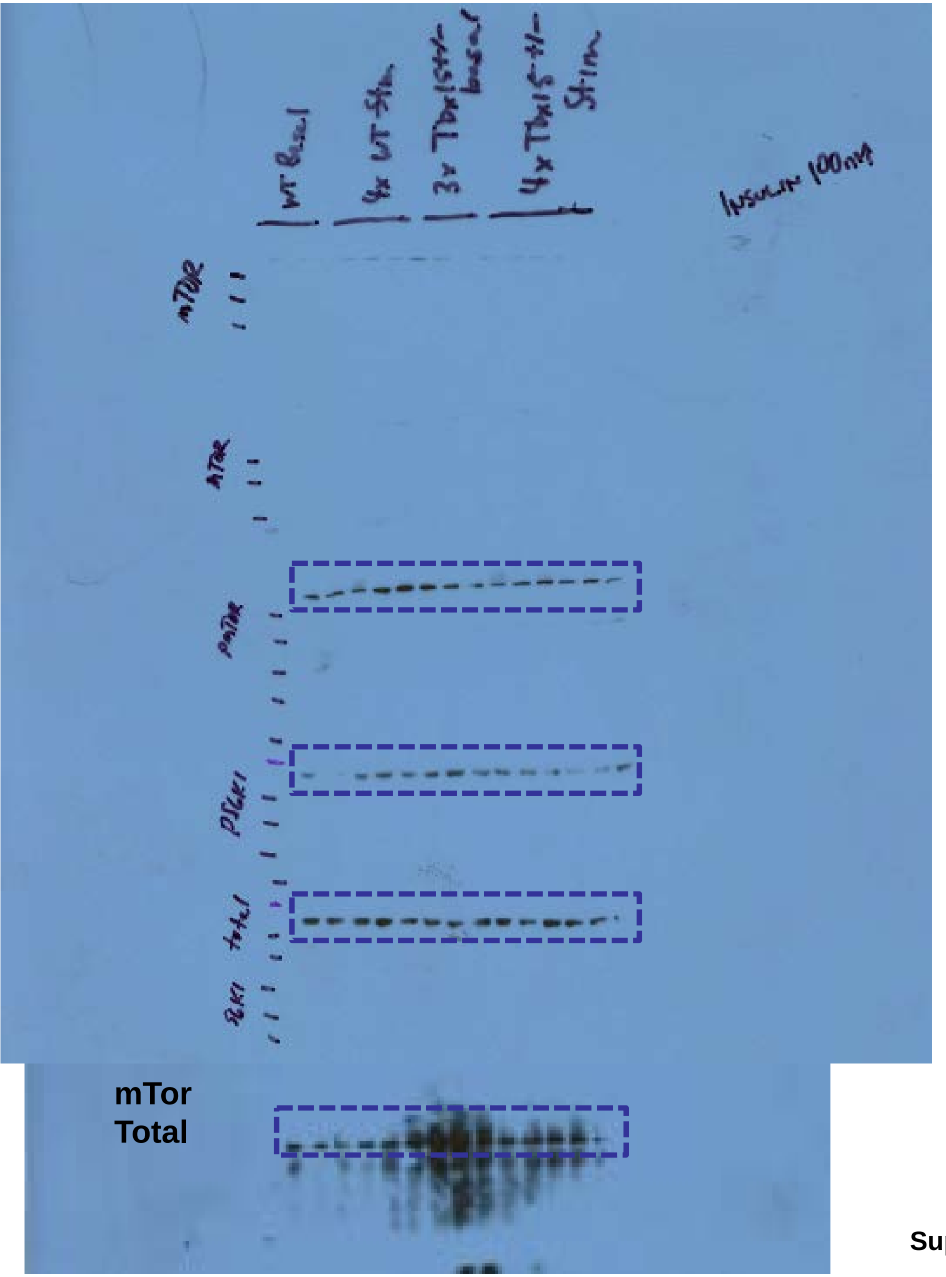

Supplementary Figure 7. Uncropped gels of key Western blot experiments

Supplementary Table 1. Sequence of qPCR Primers

Gene	Sequence
MyoD1 F	AGCACTACAGTGGCGACTCAGAT
MyoD1 R	TCCACTATGCTGGACAGGCAGT
Myf5 F	AATGCCATCCGCTACATTGAGAGC
Myf5 R	TGTCAAAGCTGCTGTTCTTTCGGG
Myogenin F	TTGCTCAGCTCCCTCAACCAGGA
Myogenin R	AGATTGTGGGCGTCTGTAGGGTCA
Myostatin F	TGGCTCAAACAGCCTGAATCCAAC
Myostatin R	TGGGTGTGTCTGTCACCTTGACTT
Baf60c F	CCGCTCAGCCATTGTCC
Baf60c R	TGATGACGTGGTTGATCACAAT
Deptor F	GCTTTGTGGTACGAGGAAGTAA
Deptor R	TGTTGTGTTGTTCTGGGATAGG
Igf2 F	GGAGCTTGTTGACACGCTTCAGTT
Igf2 R	AAGTACGGCCTGAGAGGTAGACA
mTbx15	TGTTCGCACACTGACCTTTG
mTbx15	CCAGTGCTGGAGGTGGTT
TBP	ACCCTTCACCAATGACTCCTATG
TBP	TGACTGCAGCAAATCGCTTGG
PGC1± F	TGATGTGAATGACTTGGATACAGACA
PGC1± R	GCTCATTGTTGTACTGGTTGGATATG
Rip140 F	CTCAGCTTCCTTTCCCACATAG
Rip140 R	GTCTCAGGGAATATGCTGGTTT
Myosin I F	ACCAGGCCCTTTGACCTCAAGAAA
Myosin I R	TCTTGTCGAACTTGGGTGGGTTCT
Myosin IIa F	TCACATCCAACAAGAAGCCAGAGC
Myosin IIa R	CCCTGGCTGACAAATGGGTAATCA
Myosin IIb F	AGTCCCAGGTCAACAAGCTG
Myosin IIb R	TTTCTCCTGTCACCTCTCAACA
Sdha F	ATATGGTGCAGAAGCTCGGAAGGA
Sdha R	TGTCCACATATGAGAGGGTGTGCT

Supplementary Table 2. Antibodies

Antibody	Vendor	Catalog Number
Tbx15	Open Biosystems	Custom Peptide Antibody
Tubulin	Santa Cruz Biotechnologhy	H-235
Myosin Heavy Chain I	DSHB University of Iowa	BA-D5
Myosin Heavy Chain IIa	DSHB University of Iowa	SC-71
Myosin Heavy Chain IIb	DSHB University of Iowa	BF-F3
phospho AMPK Thr172	Cell Signaling Technology	2531
AMPK	Cell Signaling Technology	2532
phospho ACC Ser79	Cell Signaling Technology	3661
ACC	Cell Signaling Technology	3662
Actin	Cell Signaling Technology	sc47778
phospho Akt S473	Cell Signaling Technology	9271
AKT	Cell Signaling Technology	9272
phospho ERK T202/Y204	Cell Signaling Technology	4377
ERK	Cell Signaling Technology	9102
phospho mTOR Ser2448	Cell Signaling Technology	2971
mTor	Cell Signaling Technology	2972
phospho p70S6K1 T389	Cell Signaling Technology	9206
p70S6K1	Cell Signaling Technology	2708
Igf2	Abcam	ab9574

Supplementary Table 3. Genes Downregulated in shTbx15 and Upregulated in pBABE-Tbx15

Gene Title	Gene Symbol	shTbx15/shSCR	pB-Tbx15/pB-Empty
coiled-coil domain containing 7	Ccdc7	0.53	8.08
killer cell lectin-like receptor, subfamily D, member 1	Klrd1	0.58	4.03
serum amyloid A 3	Saa3	0.44	2.64
collagen, type VIII, alpha 2	Col8a2	0.64	2.59
chemokine (C-C motif) ligand 17	Ccl17	0.36	2.52
kelch repeat and BTB (POZ) domain containing 11	Kbtbd11	0.30	2.48
tetraspanin 11	Tspan11	0.55	2.45
dynein, axonemal, intermediate chain 1	Dnaic1	0.60	2.44
a disintegrin and metallopeptidase domain 34	Adam34	0.62	2.42
RIKEN cDNA A730054J21 gene	A730054J21Rik	0.65	2.40
poly(A) binding protein, cytoplasmic 5	Pabpc5	0.58	2.36
RIKEN cDNA E030031F02 gene	E030031F02Rik	0.58	2.31
insulin-like growth factor 2	Igf2	0.59	2.28
ATPase, H+ transporting, lysosomal V1 subunit H	Atp6v1h	0.43	2.28
transmembrane and immunoglobulin domain containing 1	Tmigd1	0.61	2.06
transmembrane protein 132E	Tmem132e	0.62	2.00
ankyrin repeat domain 39	Ankrd39	0.65	1.92
keratin 19	Krt19	0.64	1.88
Cytochrome P450, family 2, subfamily w, polypeptide 1	Cyp2w1	0.61	1.88
pleckstrin	Plek	0.64	1.87
ST8 alpha-N-acetyl-neuraminide alpha-2,8-sialyltransferase 1	St8sia1	0.54	1.85
thymidylate synthase, pseudogene	Tyms-ps	0.49	1.84
complement component 1, r subcomponent A	C1ra /// C1rb	0.60	1.84
RIKEN cDNA 1810030J14 gene	1810030J14Rik	0.48	1.74
cytoplasmic polyadenylation element binding protein 2	Cpeb2	0.64	1.68
WD repeat domain 76	Wdr76	0.53	1.66
placenta-specific 8	Plac8	0.52	1.66
RIKEN cDNA 1700123K08 gene	1700123K08Rik	0.57	1.64
insulin-like growth factor binding protein 4	Igfbp4	0.44	1.63
solute carrier family 2 (facilitated glucose transporter), member 3	Slc2a3	0.61	1.61
Crx opposite strand transcript 1	Crxos1	0.38	1.58
phospholipase C, beta 1	Plcb1	0.64	1.58
RIKEN cDNA 1500005C15 gene	1500005C15Rik	0.64	1.57
potassium channel tetramerisation domain containing 12b	Kctd12b	0.52	1.57
piwi-like homolog 4 (Drosophila)	Piwil4	0.61	1.56
butyrophilin-like 9	Btnl9	0.39	1.54
GTP cyclohydrolase 1	Gch1	0.62	1.54
ceruloplasmin	Cp	0.59	1.50

Supplementary Table 4. Genes Upregulated in shTbx15 and Downregulated in pBABE-Tbx15

Gene Title	Gene Symbol	shTbx15/shSCR	pB-Tbx15/pB-Empty
hemoglobin alpha, adult chain 1 /// hemoglobin alpha, adult chain 2	Hba-a1 /// Hba-a2	1.59	0.34
Homeobox A10	Hoxa10	1.58	0.42
leucine rich repeat protein 1, neuronal	Lrrn1	1.70	0.47
SRY-box containing gene 4	Sox4	1.50	0.50
thiosulfate sulfurtransferase (rhodanese)-like domain containing 1	Tstd1	2.76	0.50
oxysterol binding protein 2	Osbp2	1.49	0.52
nebulette	Nebl	1.47	0.55
guanylate binding protein 2	Gbp2	1.48	0.55
regulator of calcineurin 1	Rcan1	1.43	0.56
sarcolemma associated protein	Slmap	1.74	0.58
Rho-related BTB domain containing 1	Rhobtb1	1.52	0.58
similar to Transmembrane gamma-carboxyglutamic acid protein 4 precursor (P	LOC676960 /// Prrg4	1.99	0.58
RIKEN cDNA B230216G23 gene	B230216G23Rik	1.95	0.60
major facilitator superfamily domain containing 6	Mfsd6	1.48	0.62
pleckstrin homology domain interacting protein	Phip	1.47	0.62
UDP-glucose ceramide glucosyltransferase	Ugcg	1.64	0.62
RIKEN cDNA 1600029D21 gene	1600029D21Rik	1.99	0.63
Kv channel interacting protein 4	Kcnip4	1.54	0.63
poliovirus receptor	Pvr	1.69	0.65
ring finger protein 144B	Rnf144b	1.53	0.65
beta galactoside alpha 2,6 sialyltransferase 1	St6gal1	1.60	0.66
progestin and adipoQ receptor family member VIII	Paqr8	1.83	0.66
